# TGFβ signaling sensitizes MEKi-resistant human melanoma to targeted therapy-induced apoptosis

**DOI:** 10.1038/s41419-024-07305-1

**Published:** 2024-12-21

**Authors:** Benjamin Loos, Adrian Salas-Bastos, Anna Nordin, Julien Debbache, Salome Stierli, Phil F. Cheng, Stefanie Rufli, Conrad Wyss, Mitchell P. Levesque, Reinhard Dummer, Wendy Wei-Lynn Wong, Steve Pascolo, Claudio Cantù, Lukas Sommer

**Affiliations:** 1 University of Zürich, Institute of Anatomy, Winterthurerstrasse 190, 8057 Zürich, Switzerland; 2 Wallenberg Centre for Molecular Medicine, Linköping University, 58185 Linköping, Sweden; 3 Department of Biomedical and Clinical Sciences, Division of Molecular Medicine and Virology; Faculty of Medicine and Health Sciences, Linköping University, 58185 Linköping, Sweden; 4 University of Zürich Hospital, University of Zürich, Department of Dermatology, Raemistrasse 100, 8091 Zürich, Switzerland; 5 University of Zurich, Institute of Experimental Immunology, Winterthurerstrasse 190, 8057 Zürich, Switzerland; 6 Department of Molecular Life Sciences, University of Zürich, Zürich, Switzerland; 7 Faculty of Medicine, University of Zürich, Zürich, Switzerland

**Keywords:** Skin cancer, Cell death

## Abstract

The TGFβ signaling pathway is known for its pleiotropic functions in a plethora of biological processes. In melanoma, TGFβ signaling promotes invasiveness and metastasis formation. However, its involvement in the response to therapy is controversial. While several studies have linked TGFβ signaling to elevated resistance to targeted therapy in melanoma, separate findings have indicated a favorable treatment response through TGFβ-mediated increase of cell death. We now found that the outcome of TGFβ signaling in the context of targeted therapy is dose dependent. Unlike low doses, high levels of TGFβ signal activation induce apoptosis upon simultaneous MAPK pathway inhibition, even in targeted therapy resistant melanoma cell lines. Using transcriptomic analyses, combined with genomic target identification of the critical TGFβ signaling effector SMAD4, we demonstrate that parallel activation of TGFβ signaling and MAPK pathway inhibition causes a complete switch of TGFβ target genes from promoting pro-invasive processes to fueling pro-apoptotic pathways. Investigations of underlying mechanisms identified a novel apoptosis-inducing gene signature. Functional validation of signature members highlighted a central role of the pro-apoptotic BCL2 family member *BCL2L11* (BIM) in mediating apoptosis in this condition. Using a modified, synthetic version of the *TGFB1* mRNA for intra-tumoral injections, we additionally showcase a potential therapeutic application of this treatment combination.

## Introduction

The transforming growth factor beta (TGFβ) signaling pathway has been implicated in numerous biological processes, giving rise to a diverse array of cellular reactions [1]. Besides inducing apoptosis [2–4], TGFβ signaling can influence formation of myofibroblasts [5] and can further serve as a driver for the transition from epithelial to mesenchymal (EMT) states or even stem cell like states [6–8]. In cancer, TGFβ signaling often presents the dichotomy of a “double-edged sword” [9], inhibiting proliferation in early stages [10], while promoting invasiveness and metastasis formation in later stages of tumor progression [11, 12].

In melanoma, however, the activation of TGFβ signaling has mostly been shown to promote tumorigenesis and increase invasiveness [13]. While inhibition of the TGFβ signaling pathway reduced invasive capacities in vitro and in vivo [14, 15], more metastasis formation was observed in a genetically engineered melanoma mouse model upon increased TGFβ signaling [16]. Moreover, TGFβ-dependent phenotype switching was identified as a mechanism inducing cellular states that display elevated resistance against targeted therapy approaches [17]. Increased expression of TGFβ pathway members was detected in targeted therapy resistant melanoma lines [18–20] and exposure of melanoma cell lines to TGFβ1 ligand increased their survival when subjected to targeted therapy [18, 20]. In line with this, elevated expression levels of the TGFβ signal mediator SMAD3 have been associated with resistance to MAPK pathway inhibition [21]. In contrast, other studies showed that stimulation of the TGFβ signaling pathway and parallel treatment with MAPK pathway inhibitors lead to an increase in cell death [22, 23].

As in melanoma, TGFβ signaling can elicit distinct responses during embryonic development. Melanoma arises from the melanocytic lineage, which during development originates from the neural crest [24]. Melanoma cells can harness neural crest stem cell (NCSC)-like features allowing them to reactivate migratory capacities and escape immune surveillance [25, 26]. Fate decisions of NCSCs, however, are strongly dependent on their environment [27, 28]. In particular, the effects of TGFβ signaling on neural crest cell development is highly dependent on the availability and concentration of the ligand [29, 30]. While low levels of TGFβ facilitate the differentiation of neurons from NCSCs, higher concentrations cause apoptosis [31]. Within this study, we sought to address the discrepancy in the literature on how TGFβ signaling is acting on melanoma cells in the context of MAPK pathway inhibition by considering possible dose-dependent responses. While on their own neither low nor high doses of TGFβ1 can promote cell death, high concentrations of TGFβ1 have a strong pro-apoptotic effect upon simultaneous MAPK pathway inhibition. This is associated with a drastic switch in TGFβ-induced target genes to a novel TGFβ associated pro-apoptotic gene signature, unrelated to commonly known pro-invasiveness gene sets. Finally, therapeutic upregulation of TGFβ signaling is able to create an apoptosis-promoting context in melanoma tumors in vivo, thus overcoming therapy resistance.

## Results

### Dose-dependent effects of TGFβ1 in the treatment response to targeted therapy

**Fig. 1:**
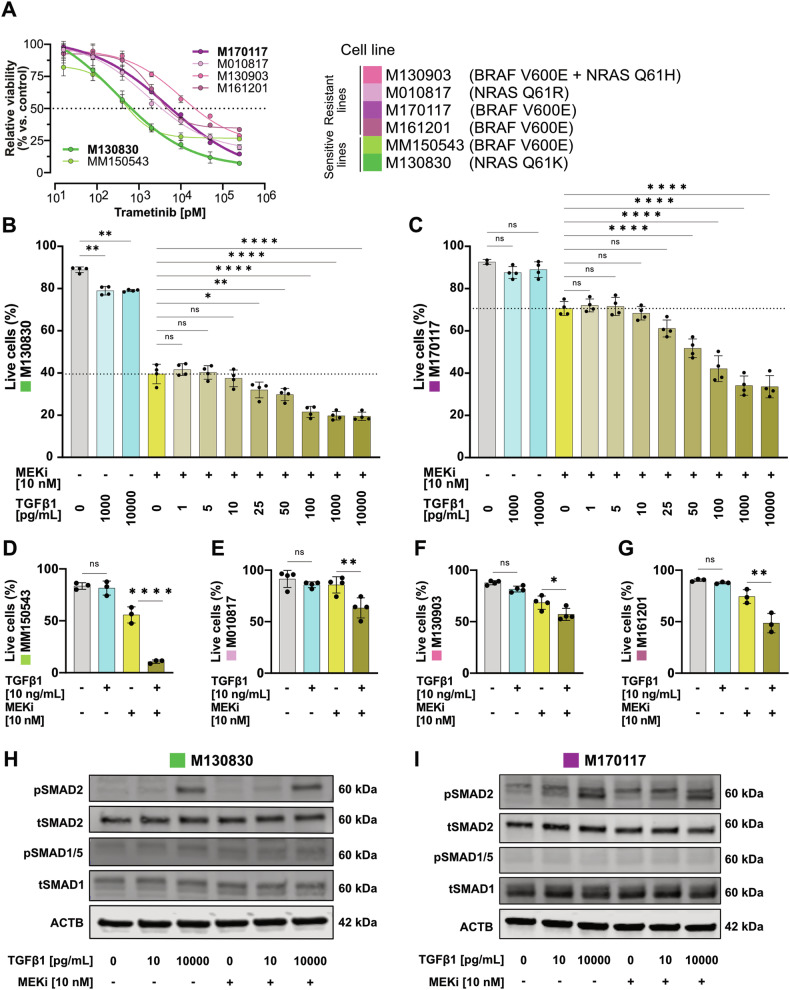
High TGFβ1 doses promote cell death upon MEKi treatment. **A** Dose-response curves of cell growth of six patient-derived melanoma cell lines treated with trametinib as determined in a colorimetric metabolic activity (MTT) assay after 72 h of treatment. Cell lines were considered sensitive with an IC50 lower than 2000 pM. Sensitive lines are shown in green and resistant lines in pink. **B**, **C** Flowcytometry quantification of Annexin V negative cells (“Live cells”) after 72 h exposure to various concentrations of TGFβ1 and/or 10 nM trametinib (MEKi) in a putative “sensitive” (**B**) and a “resistant” (**C**) melanoma cell line. **D**–**G** Live cell quantification upon TGFβ1 (10 ng/mL) and/or MEKi (10 nM) treatment in another “sensitive” melanoma cell line (**D**) and three further “resistant” cell lines (**E**–**G**). Experiments were performed in 3–4 independent replicates. *P*-values were calculated by ordinary one-way ANOVA and multiple comparisons for selected pairs with *p < 0.05, **p < 0.01, ***p < 0.001 and ****p < 0.0001. **H**, **I** Western blots for phosho-SMAD2 (pSMAD2), total SMAD2 (tSMAD2), phosho-SMAD1/5 (pSMAD1/5), total SMAD1 (tSMAD1) and ACTB from whole cell lysates of a sensitive (**H**) and a resistant (**I**) melanoma cell line after 1 h of TGFβ1 / MEKi combinatorial treatment.

To evaluate how TGFβ signaling influences melanoma cells in absence or presence of MAPK pathway inhibition, we first tested human melanoma cell lines harboring either oncogenic *NRAS*^Q61X^ or *BRAF*^V600E^ mutations for their sensitivity to trametinib, a selective MEK1/MEK2 inhibitor [32] referred later as MEKi. Cells were categorized as sensitive or resistant to trametinib based on their response over 72 h (Fig. 1A). The viability of the cells was measured using a colorimetric assay to determine the half maximal inhibitory concentration (IC50) for each line. Western blot analysis for phosphorylated ERK (pERK) confirmed the downregulation of the MAPK signaling pathway in both sensitive and resistant cell lines upon trametinib treatment (Supplementary Fig. 1A).

To explore the interaction between TGFβ signaling and therapy response, we further investigated two of the initial 6 cell lines, designated respectively as “sensitive” (M130830) and “resistant” (M170117) to MEKi-induced cell death. Both were treated with varying doses of TGFβ1 in presence or absence of trametinib. Cell death, inferred by Annexin V positivity using flow cytometry, showed no significant increase at lower TGFβ1 doses (1–10 pg/ml) combined with MEKi compared to MEKi alone (Fig. 1B, C). Likewise, high dose TGFβ1 stimulation (≥1000 pg/ml) by itself did not or only slightly enhance cell death. However, upon co-treatment with MEKi, high TGFβ1 concentrations (≥25 pg/ml) significantly increased the number of dead cells (Fig. 1B, C). A robust increase in cell death upon double treatment, compared to MEKi alone, was confirmed in other sensitive and resistant cell lines (Fig. 1D–G).

These results indicate that TGFβ signaling markedly affects melanoma cell responses to targeted therapy in a dose-dependent manner. The distinct increase in apoptosis in the double treatment condition, compared to TGFβ1 alone, hints at a possible alteration in TGFβ-induced signaling under these conditions. Although TGFβ1 is thought to mediate signaling mostly through SMAD2/3 phosphorylation, it can activate the SMAD1/5/8 signaling branch as well [33, 34]. However, in the context of TGFβ1 and MEKi treatment, only SMAD2 phosphorylation, but not SMAD1/5, was observed (Fig. 1H, I). Moreover, pSMAD2 levels were not changed upon TGFβ1 + MEKi co-treatment as compared to TGFβ1 alone, suggesting that the altered cellular response is not due to modulation of the TGFβ signaling strength. Likewise, TGFβ1 did not alter pERK levels in MEKi-treated melanoma cells (Supplementary Fig. 1A), while there was a slight increase in pERK levels upon TGFβ1 stimulation in some of the cell lines (Supplementary figure 1A), consistent with previous findings that TGFβ can activate the MAPK pathway in a context-dependent manner [35–37].

In our assay, *NRAS*^Q61X^ and *BRAF*^V600E^ mutant cell lines showed comparable responses to TGFβ1 + MEKi double treatment. To further elaborate whether differences in driver mutations would influence this effect, three melanoma cell lines, which do neither carry a *BRAF*^*V600E*^ nor a *NRAS*^*Q61X*^ driver mutation (MM140325, M100916 and MM170522, also referred to as *BRAF/NRAS*-wt lines), were additionally investigated. In these *BRAF/NRAS*-wt lines, the MAPK pathway was activated, as shown by abundant phosphorylated ERK levels, which were downregulated upon MEKi treatment (Supplementary Fig. 1B–D). Like the *BRAF*^*V600*^ and *NRAS*^*Q61*^ mutated lines, cell death was increased in the double treatment condition compared to the single treatments with TGFβ1 or MEKi alone (Supplementary Fig. 1E–G). Interestingly, however, the *BRAF/NRAS*-wt lines showed a higher susceptibility to TGFβ1 treatment alone, in contrast to the *BRAF*^*V600*^ and *NRAS*^*Q61*^ mutated lines. Importantly, increased cell death upon TGFβ1 + MEKi treatment was observed in all cell lines independently of their driver mutation.

### Rescue effect by pan caspase inhibitor ZVAD and presence of cleaved caspase 3 and PARP confirm apoptosis as mode of cell death

**Fig. 2:**
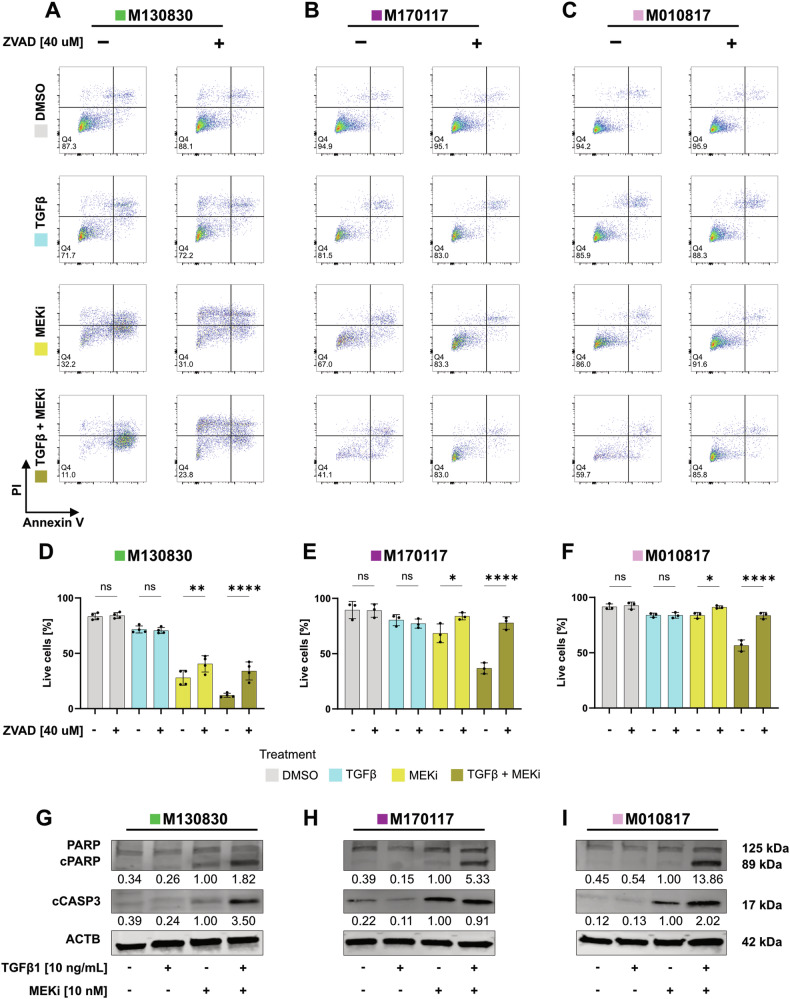
Apoptosis is the mode of cell death induced by concomitant TGFβ1 and MEKi treatment in melanoma cells. **A**–**C** Representative FACS plots of the three melanoma cell lines M130830 (**A**), M170117 (**B**) and M010817 (**C**) treated with combinations of TGFβ1 (10 ng/mL), MEKi (10 nM) and ZVAD (40 µM) for 72 h as indicated and stained with Annexin V and Propidium Iodide (PI) solution. **D**–**F** Live cell quantification of the FACS staining from (**A**–**C**). Experiments were performed in 3 independent replicates. *P*-values were calculated by one-way ANOVA and multiple comparisons for selected pairs with *p < 0.05, **p < 0.01, ***p < 0.001 and ****p < 0.0001. **G**–**I** Western blots for PARP, cCASP3, and β-ACTIN (ACTB) from whole cell lysates of one sensitive, M130830 (**G**) and two resistant melanoma cell lines, M170117 (**H**) and M010817 (**I**), treated with combinations of TGFβ1 and MEKi for 48 h as indicated. cCASP3 and cPARP levels were quantified relative to the loading control with the MEKi only condition set to 1 and shown below the blots.

To address whether TGFβ1 + MEKi-induced cell death occurs through apoptosis, we included the pan caspase inhibitor ZVAD in our cell culture experiments. Both in the MEKi only and the TGFβ1 + MEKi condition, cell death was reduced when ZVAD was administered, as shown by FACS staining for Annexin V and Propidium Iodide (PI) (Fig. 2A–F). For the sensitive cell line M130830, only a partial rescue was achieved by ZVAD addition, suggesting that in this cell line cell death occurred through apoptosis as well as other mechanisms. In contrast, an almost complete rescue effect was observed for the cell lines M170117 and M010817. The presence of cleaved caspase 3 (cCASP3) and of the 89 kDa fragment of cleaved poly(ADP-ribose)-polymerase (PARP) [38] further validates apoptosis as the main mode of cell death in response to the combined TGFβ1 + MEKi treatment (Fig. 2G–I).

### Transcriptome analyses reveal MEKi-dependent switch of TGFβ1-induced gene expression

To investigate the downstream effectors of context-dependent TGFβ signaling, bulk RNA sequencing (RNAseq) was conducted on human melanoma cell lines under four different conditions: control (DMSO), TGFβ1 only, MEKi only, and TGFβ1 + MEKi. Samples were collected for sequencing at 20 h post-treatment, which was identified as the timepoint at which cell numbers started to decline considerably upon TGFβ1 + MEKi double treatment (Supplementary figure 1H).

**Fig. 3:**
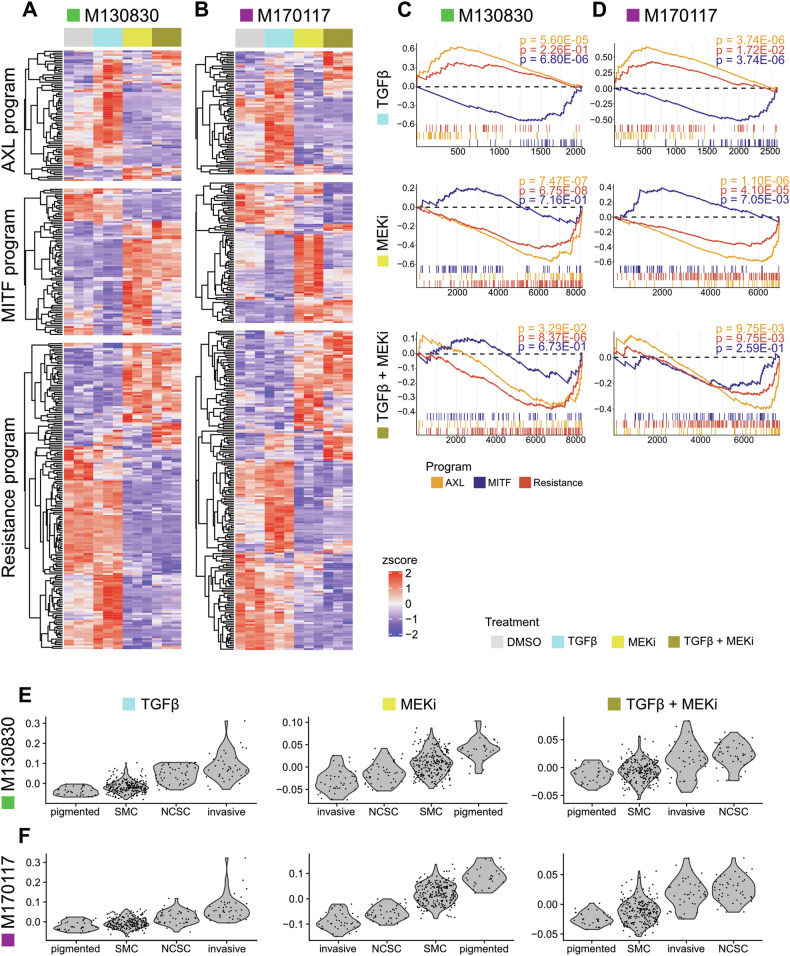
Double treatment induces a distinctive gene expression signature as compared to the individual treatments. **A**, **B** Heatmaps of bulk RNAseq data of a “sensitive” (**A**) and a “resistant” (**B**) human melanoma cell line, representing differentially expressed genes (*p* adjusted < 0.05) that are part of previously defined [36, 39] melanoma programs: AXL, MITF and the resistance program. The expression values are z-score normalized per row and genes are clustered using hierarchical clustering with euclidean distance. **C**, **D** Gene set enrichment analyses (GSEAs) of each program (*p* adjusted value < 0.05) under each condition compared to the vehicle control (DMSO) in a sensitive (**C**) and a resistant cell line (**D**). Running Enrichment Score is shown on the Y-axis, gene number on the *x*-axis. **E**, **F** Scores of TGFβ1, MEKi or TGFβ1 + MEKi treatment signatures represented in cell subpopulations previously identified by single cell RNAseq analysis in a xenograft melanoma model during BRAFi/MEKi treatment [37]. Violin plots show cell score distribution within each cell population in a sensitive (**E**) and a resistant (**F**) cell line. “SMC” = starved-like melanoma cell state. “NCSC” = neural crest stem cell state.

To explore differences among treatment conditions, we compared RNAseq data with established melanoma signatures. Tirosh and colleagues defined proliferative (MITF program), invasive (AXL program), and resistant (Tirosh “Resistance” program) melanoma cell states using single-cell RNAseq (scRNAseq) data from patients [39]. Utilizing these predefined programs as references, we found differentially expressed genes in all treatment conditions for both cell lines, illustrated in cell line-specific heatmaps (Fig. 3A, B). Despite differing in MEKi sensitivity, both cell lines exhibited similar expression patterns across all three programs. The TGFβ1-only condition showed an upregulation of AXL program genes, while MITF program genes remained low. “Resistance program” associated genes also showed increased expression, highlighting the role of TGFβ in resistance development. Strikingly, however, several genes of the “AXL” or “Resistance” programs were predominantly downregulated in the double treatment (TGFβ1 + MEKi) condition. This strongly suggests that MAPK pathway inhibition hinders the induction of a substantial number of TGFβ downstream target genes. Gene set enrichment analysis (GSEA) confirmed these observations, revealing a strong correlation to the AXL program in the TGFβ1-only condition and a substantial anti-correlation in the double treatment for both cell lines (Fig. 3C, D). This confirms a MEKi dependent blockade of TGFβ1-induced gene expression patterns associated with invasiveness. Further analysis was done by comparing the data with a dataset from Rambow et al. [40], which characterized melanoma cell states during treatment. Gene signatures derived from our treatment conditions were evaluated in these reported cellular states (Fig. 3E, F). While the TGFβ1-only condition correlated mainly with the ‘invasive’ state, MEKi alone aligned best with the ‘pigmented’ state. Remarkably, the double treatment signature correlated mostly with the ‘NCSC’ state, indicating a distinct cell identity in this condition.

SMAD specific E3 ubiquitin protein ligase 2 (*SMURF2*) has previously been identified as a negative regulator of TGFβ signal activity and found to be upregulated in response to concomitant TGFβ1 and MEKi treatment [23]. Our data confirm an induction of *SMURF2* expression upon stimulation with TGFβ1 alone, but not in combination with MEKi (Supplementary Fig. 1I). Despite this discrepancy, we did not observe an increase in pSMAD2 levels in the double-treatment condition (Fig. 1H, I), suggesting that the increased apoptosis observed in our experiments is independent of changes in *SMURF2* and pSMAD2 levels.

Moreover, in line with a previous study identifying Twist Family BHLH Transcription Factor 1 (*TWIST1*) as an anti-apoptotic modulator in melanoma [22], we observed downregulation of *TWIST1* upon combined treatment with TGFβ1 and MEKi. However, unlike the earlier report, we found *TWIST1* levels to be decreased also upon TGFβ1 stimulation, independently of MEKi treatment (Supplementary Fig. 1J), indicating that *TWIST1* is not part of the apoptosis-inducing program specific to TGFβ1 + MEKi treatment.

### Identification of potential target genes implicated in TGFβ1 + MEKi mediated apoptosis

**Fig. 4:**
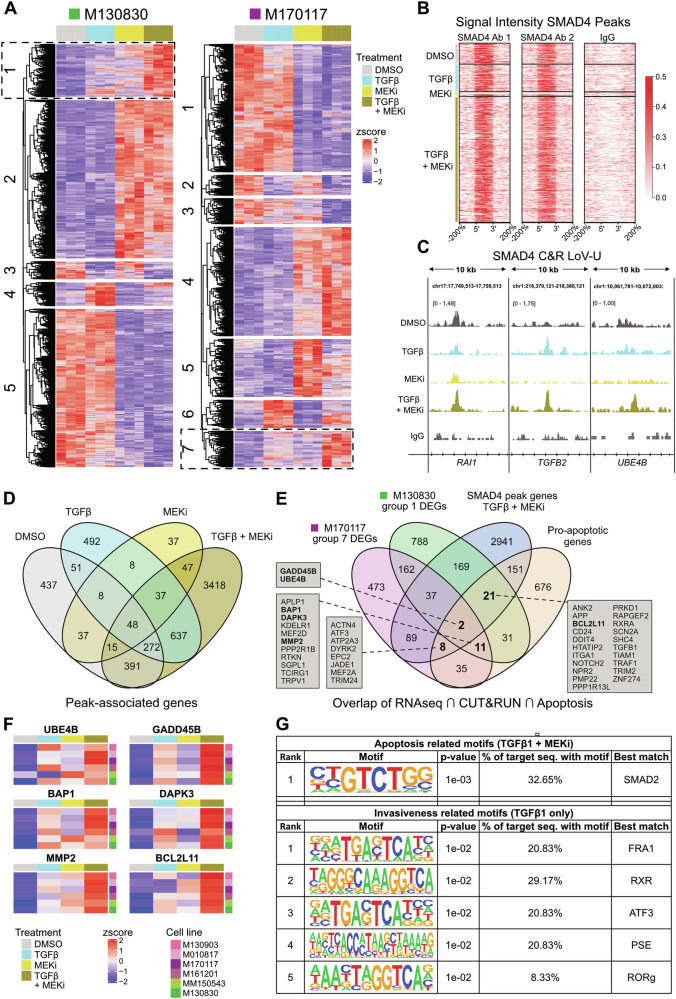
Comparison of bulk RNAseq and SMAD4 CUT&RUN data reveals potential mediators of elevated apoptosis effect. **A** Individual heatmaps of bulk RNAseq data of M130830 and M170117 cells, representing differentially expressed genes (*p* adjusted < 0.05; *z*-score normalized by row) for each treatment condition (DMSO, TGFβ1, MEKi or TGFβ1 + MEKi). Groups of gene clusters that were taken into further analyses are indicated by dashed boxes. **B** Signal intensity plots of SMAD4 CUT&RUN showing reproducible peak regions for two SMAD4 antibodies used in each condition (left + middle panel), plus IgG negative control (right panel) in the M170117 cell line. Peak size is scaled (each peak’s length is 100%) to allow comparison of signal intensity across target loci genome-wide. The *x*-axis includes genomic intervals equivalent to 4 times the size of each peak spanning the region at the 5′ upstream (−200%) and the 3′ downstream (200%) of the peak. **C** Examples of CUT&RUN SMAD4 data as visualized in Integrative Genome Viewer, showing a peak called in all conditions (left, *RAI1*), one present under TGFβ1 stimulation (center, *TGFB2*) and one only present under TGFβ1 + MEKi treatment (right, *UBE4B*). Shown tracks are merged from the two replicates and scaled to signal per million reads. **D** Venn diagram showing the overlap of peak associated genes across treatments, as annotated by GREAT (http://great.stanford.edu/public/html/index.php). **E** Venn diagram showing overlaps of differentially expressed genes (DEGs) in the TGFβ1 + MEKi condition for the cell lines M130830 (group 1 left heatmap, indicated by dashed box) and M170117 (group 7 right heatmap, indicated by dashed box) as well as unique peak associated genes for the TGFβ1 + MEKi condition identified by CUT&RUN SMAD4 for the M170117 cell line (3418 genes) and a pre-defined pro-apoptotic gene signature (GO:0006915—“Apoptotic processes” subtracted by GO:0043066—“Negative regulation of apoptosis”). Subsets of interest are highlighted by bold letters and were used to determine the TGFβ1 + MEKi-specific apoptosis signature. Genes lists of each subset are shown in grey boxes. **F** Heatmaps of six selected genes showing gene expression in the four different treatment conditions DMSO, TGFβ1, MEKi and TGFβ1 + MEKi for six human melanoma cell lines. Expression values are *z*-score normalized per row. **G** Motif enrichment analysis (HOMER) performed on SMAD4 CUT&RUN dataset for motifs detected in TGFβ1 + MEKi condition (Apoptosis related motifs) or TGFβ1 only condition (Invasiveness related motifs). Refined input genes were selected through overlay of original SMAD4 CUT&RUN data with bulk RNAseq dataset and pro-apoptosis or pro-invasiveness gene lists, respectively.

To identify target genes of TGFβ signaling that could potentially mediate apoptosis in the TGFβ1 + MEKi context, we sought to combine transcriptomic analysis with the characterization of DNA binding motifs of SMAD4, the common mediator of canonical TGFβ signaling [41], through CUT&RUN sequencing. To this end, we first conducted individual analysis of bulk RNAseq data for each cell line, comparing the four treatment conditions. Unsupervised clustering of the differentially expressed genes (DEGs) retrieved groups (indicated by numbers) with distinctive expression patterns (Fig. 4A). In both the MEKi-sensitive (M130830) and the MEKi-resistant (M170117) cell lines a set of highly upregulated genes was revealed in the double treatment (TGFβ1 + MEKi) condition, marked by dashed boxes in Fig. 4A. GO term analysis for the gene clusters of interest did not retrieve any enrichment for pathways related to cell death, suggesting that potential apoptosis mediators in this context may be novel.

In parallel, SMAD4 CUT&RUN sequencing was performed for the M170117 cell line across the four treatment conditions using two different SMAD4 antibodies. Overlaps of both antibodies identified reproducible peaks (Fig. 4B). Notably, the double treatment condition exhibited the most peaks, while the MEKi-only condition had the fewest. Some peaks were common across conditions (e.g., *RAI1*), while others were exclusive to one condition (e.g., *UBE4B*) (Fig. 4C). Among the uniquely identified peaks, some were predicted to influence gene expression of targets like *GADD45B* and *BCL2L11* from distant DNA sites (Supplementary Fig. 2A). Peak annotations for SMAD4 binding were mainly in intergenic or intronic DNA regions, distributed similarly across different treatment conditions (Supplementary Fig. 2B).

Software-based peak-to-gene annotation showed that the majority of SMAD4-bound genes in the double treatment (TGFβ1 + MEKi) condition were unique to that specific context (3418 out of 4865) (Fig. 4D). This underscores the distinct involvement of SMAD4 in the TGFβ signaling pathway under the dual treatment scenario. Similarly, unique gene sets were also identified in other treatment conditions: DMSO (437 out of 1259), TGFβ1 only (492 out of 1553), and MEKi only (37 out of 237), indicating the pivotal role of SMAD4 as a transcriptional mediator orchestrating TGFβ signaling outcomes in diverse contexts.

Next, we integrated bulk RNAseq with SMAD4 CUT&RUN data to identify potential drivers of apoptosis induction in the double treatment condition. This involved overlapping uniquely upregulated genes from cell lines M130830 and M170117 in the double treatment condition with genes identified solely in this condition from the SMAD4 CUT&RUN data and a gene set of putative pro-apoptotic genes (Fig. 4E). This analysis pinpointed two main potential mediators of apoptosis specific to TGFβ1 + MEKi double treatment: Growth arrest and DNA damage inducible beta (*GADD45B*) and Ubiquitin conjugation factor E4B (*UBE4B*). Further intersections identified additional genes potentially important in enhancing apoptosis. Three notable intersections were:

1. M170117 DEGs, SMAD4 CUT&RUN peak genes, and pro-apoptotic genes, revealing 8 gene candidates.
2. M130830 DEGs, SMAD4 CUT&RUN peak genes, and pro-apoptotic genes, identifying 21 gene candidates.
3. M170117 DEGs, M130830 DEGs, and pro-apoptotic genes, unveiling 11 gene candidates.

The full lists of genes from these Venn diagram intersections of interest (Fig. 4E) encompassed a total of 42 genes, which we defined as a novel apoptosis gene signature related to combinatorial TGFβ1 + MEKi treatment in melanoma.

Literature research supported the involvement of several genes in apoptotic processes, including BRCA1 associated protein-1 (*BAP1*) [42, 43], Death associated protein kinase 3 (*DAPK3*) [44, 45], Matrix metalloproteinase-2 (*MMP2*) [46–48], and BCL-2-like protein 11 (*BCL2L11*) also known as BIM [49, 50]. Of note, expression pattern analysis of these genes across all treatment conditions consistently showed high expression in the double treatment condition (Fig. 4F). A similar expression pattern was also found for most of the other genes of our newly defined apoptosis gene signature (Supplementary Fig. 2C). Western blot analysis confirmed increased BCL2L11 protein levels in the double treatment condition compared to MEKi treatment alone (Supplementary Fig. 2D, E).

To address the regulatory mechanisms that might govern the shift from TGFβ signaling-induced pro-invasive to pro-apoptotic gene regulation, transcription factor motifs within identified SMAD4 binding regions were explored. As input, refined lists based on overlaps of bulk sequencing and CUT&RUN data were used, combined with known gene sets related to apoptosis (Fig. 4E; 31 genes) or invasiveness (Supplementary figure 2F; 20 genes). While the SMAD2 motif was the only motif identified in the TGFβ1 + MEKi double treatment condition, multiple motifs were found in the TGFβ1 only condition such as FOS Like 1, AP-1 transcription factor subunit (*FRA1*), retinoid X receptor (*RXR*), Activating transcription factor 3 (*ATF3*), small nuclear RNA activating complex polypeptide 1 (*PSE*), and RAR-related orphan receptor C (*RORg*) (Fig. 4G). Although only few genes were taken as input for this analysis, the data suggest that upon concomitant TGFβ1 stimulation and MEK inhibition, SMAD4 switches its target motifs and primarily acts in association with SMAD2 to induce expression of a specific subset of apoptosis mediators.

### TGFβ1 + MEKi mediated apoptosis can be partially rescued by downregulation of *UBE4B*, *BAP1* and *BCL2L11*

**Fig. 5:**
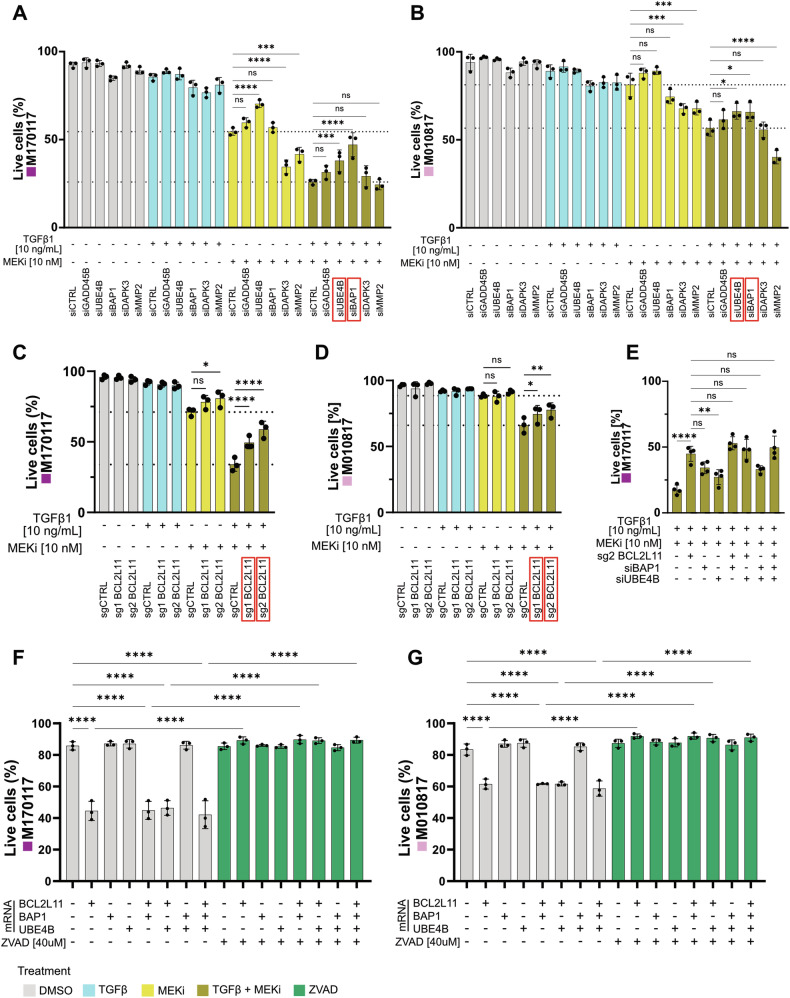
Downregulation of *UBE4B*, *BAP1* and *BCL2L11* partly rescues apoptotic effect mediated by TGFβ1 + MEKi stimulation. **A**, **B** Live cell quantification of MEKi-resistant M170117 (**A**) and M010817 (**B**) cells, after siRNA mediated downregulation of the indicated genes and treatment with DMSO or combinations of 10 ng/mL TGFβ1 or 10 nM MEKi (trametinib) for 72 h. **C**, **D** Live cell quantification of M170117 (**C**) and M010817 (**D**) upon genetic knock-out of *BCL2L11* with two different sg sequences and one non-targeting control sequence, after treatment with DMSO or combinations of 10 ng/mL TGFβ1 or 10 nM MEKi (trametinib) for 72 h. Experiments in **A**–**D** were performed in three independent replicates. Conditions with a significant rescue effect upon TGFβ1 + MEKi treatment are highlighted by red frames. **E** Live cell quantification of M170117 cells after combinatorial downregulation of *BCL2L11*, *BAP1* and/or *UBE4B* and treatment with 10 ng/mL TGFβ1 and 10 nM MEKi (trametinib) for 72 h. **F**, **G** Live cell quantification of M170117 (**F**) and M010817 (**G**) cells, 24 h after transfection with in total 200 ng of *BCL2L11*, *BAP1* and/or *UBE4B* mRNA as well as parallel treatment with 40 µM ZVAD as indicated below. Experiments in **E**–**G** were performed in four, respectively three independent replicates. *P*-values in all graphs were calculated by one-way ANOVA and multiple comparisons for selected pairs with *p < 0.05, **p < 0.01, ***p < 0.001 and ****p < 0.0001.

To functionally validate the core of our apoptosis gene signature (Fig. 4F) we conducted siRNA-mediated knockdown experiments on the two MEKi-resistant cell lines M170117 and M010817. To evaluate the involvement of these genes in TGFβ1 + MEKi mediated apoptosis, cells were treated with TGFβ1 and/or MEKi and apoptosis was quantified through Annexin V staining followed by flow cytometry (Fig. 5A, B). The efficiency of siRNA-mediated knockdowns was validated by qRT-PCR (Supplementary Fig. 3A, B). Due to low knockdown efficiencies of *BCL2L11*, a CRISPR-Cas9-mediated knock-out approach was chosen for this gene. In both cell lines (M170117 and M010817), *BCL2L11* knock-out was performed independently using two different guide sequences and validated by western blot (Supplementary figure 3C). The generated *BCL2L11*-KO cell lines were treated with all four treatment combinations and apoptosis was quantified by Annexin V staining and flow cytometry (Fig. 5C, D). In untreated control cells and in TGFβ1-treated cells, survival was generally high and not affected by decreased expression of target genes. Similarly, apart from siUBE4B-treatment in one cell line, inactivation of these genes did not rescue MEKi-induced cell death in melanoma cells. Also, upon TGFβ1 + MEKi double treatment, apoptosis was not rescued or, in some cases, was even enhanced following downregulation of *GADD45B*, *DAPK3*, or *MMP2*. In contrast, a robust apoptosis rescue effect was observed in both cell lines when *BAP1* was downregulated. Similarly, apoptosis induction was rescued, although to a lesser extent, upon downregulation of *UBE4B*. Finally, in both cell lines and with both guide sequences, *BCL2L11* knock-out significantly rescued apoptosis in the double treatment condition compared to control. Of note, for none of the genes cellular survival could be fully restored upon downregulation, indicating that the apoptotic effect is likely orchestrated through an interplay of several genes. However, investigations of combinatorial downregulations of *BCL2L11*, *BAP1* and/or *UBE4B* did not further increase the rescue effect achieved by downregulation of BCL2L11 alone in M170117 cells upon TGFβ1 + MEKi treatment (Fig. 5E). These data are consistent with the idea that BCL2L11, BAP1 and UBE4B might act through the same mode of action, with BCL2L11 being downstream of BAP1 and UBE4B.

To address whether upregulation of these three genes would promote apoptosis, the cell lines M170117 and M010817 were transfected with mRNA coding for *BAP1*, *UBE4B* and/or *BCL2L11*. Increased protein expression after each mRNA transfection was validated by western blot (Supplementary Fig. 3D). In both cell lines, apoptosis as assessed by Annexin V/PI staining and flow cytometry was consistently elevated in all conditions involving *BCL2L11* mRNA. In line with the previous combinatorial downregulation experiments, the upregulation of *BAP1* and *UBE4B* did not enhance apoptosis, even when combined with *BCL2L11* (Fig. 5F, G). In sum, these results confirm a key role of *BCL2L11* in mediating apoptosis specifically in the TGFβ1 + MEKi treatment condition.

### Apoptosis signature of TGFβ1 + MEKi co-treatment cannot be used as a predictive marker for successful targeted therapy

**Fig. 6:**
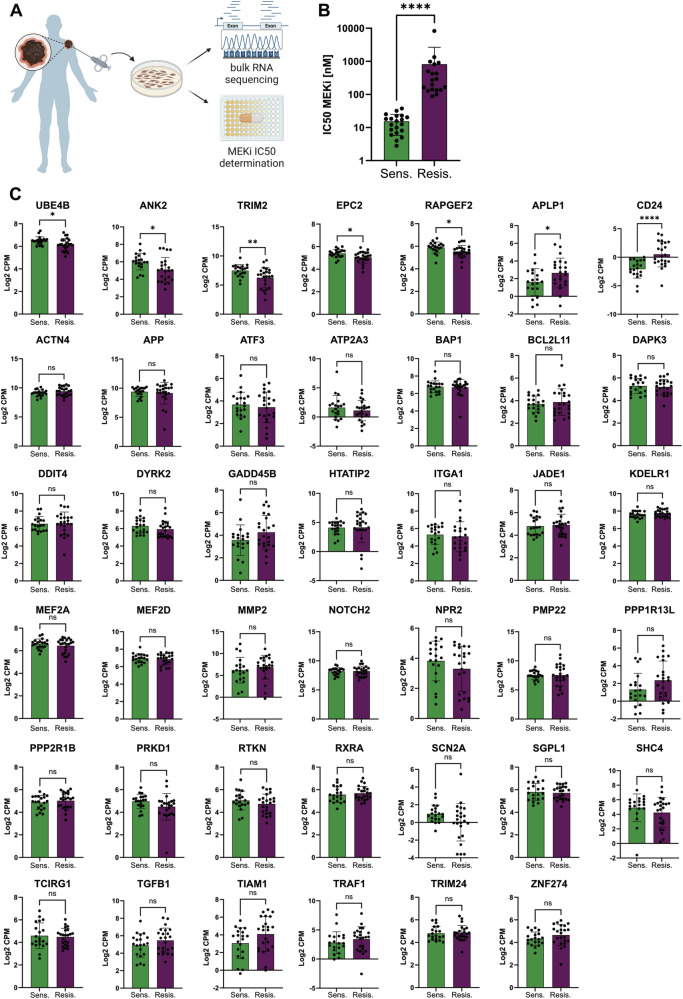
Apoptosis signature genes cannot be used as predictive markers for favorable treatment with targeted therapy. **A** Schematic setup of patient-derived melanoma cell line processing. Melanoma cell biopsies were cultured in vitro before bulk RNAseq and the half maximal inhibitory concentration (IC50) for MEKi was determined by MTT assays. **B** Representation of MEKi IC50 values for the top 20 and bottom 20 patient-derived melanoma cell lines, grouped accordingly. *P*-values were calculated by Mann-Whitney test with *p < 0.05, **p < 0.01, ***p < 0.001 and ****p < 0.0001. **C** Bar plots showing correlation of MEKi sensitive and MEKi resistant cell lines with average gene expression of all genes of the TGFβ1 + MEKi-associated apoptosis signature. *P*-values were calculated by unpaired *t*-test with *p < 0.05, **p < 0.01, ***p < 0.001 and ****p < 0.0001.

To assess if the genes identified in the novel apoptosis signature could serve as predictive markers for a favorable response to targeted therapy, we examined their baseline expression in patient-derived, treatment-naïve melanoma cell lines derived from primary and metastatic lesions. The transcriptomes of these cell lines were obtained through bulk RNAseq in an untreated state (baseline). Additionally, their sensitivity to MEKi was determined, categorizing them into MEKi-sensitive (bottom 20 IC50 values) and MEKi-resistant (top 20 IC50 values + 3 additional IC50 N/A) groups (Fig. 6A, B). The gene expression (FPKM) of each of the 42 apoptosis signature genes was determined for each selected cell line. The average expression for each group (MEKi sensitive vs. resistant) is presented in a bar chart for each gene (Fig. 6C).

While the majority of apoptosis signature genes showed similar expression levels at baseline between MEKi-sensitive and MEKi-resistant melanoma cell lines, a few genes were found to be elevated in sensitive lines, including *UBE4B*, Ankyrin-2 (*ANK2*), tripartite motif-containing protein 2 (*TRIM2*), enhancer of polycomb homolog 2 (*EPC2*), and rap guanine nucleotide exchange factor 2 (*RAPGEF2*). In contrast, two genes, Amyloid Beta Precursor Like Protein 1 (*APLP1*) and CD24 molecule (*CD24*), were elevated in MEKi-resistant lines at baseline. These findings suggest that the expression of most of the apoptosis signature genes is increased in response to concomitant treatment with MEKi and TGFβ1 and may, thus, not serve as predictive marker for effective targeted therapy before treatment.

### mRNA vector-based TGFβ1 delivery sensitizes MEKi-resistant human melanoma to targeted therapy in vivo

**Fig. 7:**
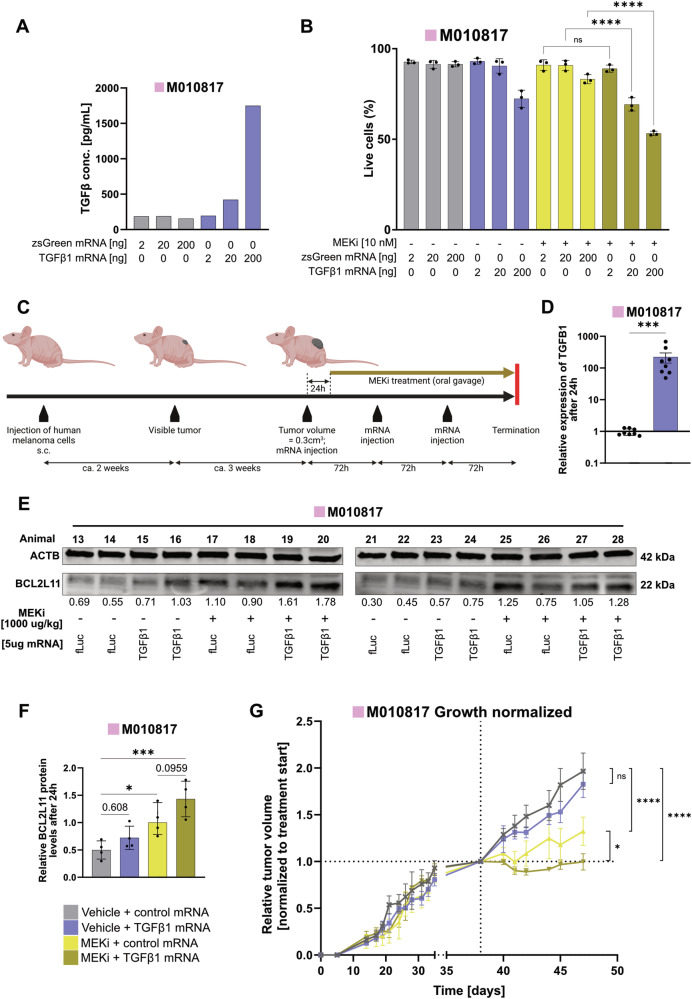
Application of *TGFB1* mRNA in combination with MEKi increases apoptosis in vitro and reduces growth of xenograft tumors in nude mice. **A** Levels of human TGFβ1 in the supernatant of M010817 cells 48 h after transfection with increasing doses of *TGFB1* mRNA or control (zsGreen) mRNA detected by ELISA. **B** Live cell quantification upon MEKi treatment (10 nM) and transfection with increasing doses of *TGFB1* mRNA in M010817 cells 72 h after transfection. Experiments were performed in 3 independent replicates. *P*-values were calculated by one-way ANOVA and multiple comparisons for selected pairs. **C** Schematic overview of the patient-derived xenograft setup indicating the timeline of tumor growth and applications of oral treatments with trametinib as well as intra-tumoral TGFβ1 or control (fLuc) mRNA injections. **D** Validation of exogenous *TGFB1* expression by RT-qPCR 24 h after transfection with *TGFB1* mRNA in M010817 xenograft tumors. *P*-values were calculated by non-parametric Mann-Whitney test. **E** Western blots for BCL2L11 and β-ACTIN (ACTB) from M010817 xenograft tumor lysates 24 h after injection with *TGFB1* mRNA and/or combinatorial treatment with MEKi (trametinib) Quantifications of BCL2L11 levels are shown below, normalized to the loading control ACTB and to the MEKi only condition (average of 2 animals per blot). **F** Quantification of BCL2L11 protein levels by western blot in M010817 xenograft tumor lysates 24 h after injection with *TGFB1* mRNA and/or combinatorial treatment with MEKi (trametinib). *P*-values were calculated by one-way ANOVA and multiple comparisons for selected pairs. **G** Growth curve of M010817 xenograft tumors in nude mice treated with MEKi or vehicle and/or *TGFB1* or control (fLuc) mRNA. *P*-values were calculated by two-way ANOVA and multiple comparisons for selected pairs. For all graphs, *p*-values are shown by numbers or with *p < 0.05, **p < 0.01, ***p < 0.001 and ****p < 0.0001.

mRNAs offer a safe and efficient way to produce therapeutic proteins inside the human body [51]. Immunosilent synthetic mRNA is made by in vitro transcription of a template DNA in the presence of methyl-1 pseudouridine triphosphate instead of uridine triphosphate. The resulting synthetic mRNA escapes sensing by RNA-specific immune sensors. Therefore, we used this modified mRNA-based vector system to validate the anti-tumor effects of increased TGFβ signaling in conjunction with MEKi in vivo. In a xenograft experiment, MEKi-resistant M010817 cells were injected into immunocompromised athymic nude mice. TGFβ signaling activation was achieved through repeated intra-tumoral injections of a synthetic mRNA encoding a mutated version of *TGFB1*, resulting in the expression of immediately active TGFβ1 ligand. Prior to the in vivo experiment, the mRNA transfection efficiency was tested in vitro using zsGreen coding mRNA and flow cytometry analyses (Supplementary Fig. 4A). The expression of TGFβ1 ligand upon in vitro *TGFB1* mRNA transfection was quantified by ELISA (Fig. 7A). Cell death assessment confirmed that *TGFB1* mRNA transfection, combined with MEKi treatment, resulted in an increased apoptosis effect as compared to control settings and MEKi treatment alone (Fig. 7B), similar to the treatment with recombinant TGFβ1 ligand (Fig. 1).

In the xenograft setup, the MEKi-resistant melanoma cell line was injected subcutaneously, and tumors were allowed to grow to approximately 300 mm^3^ before initiating three intra-tumoral injections of *TGFβ1* mRNA along with daily oral MEKi treatment (Fig. 7C). To confirm the expression of *TGFβ1* as well as the apoptosis marker BCL2L11, a short-term xenograft tumor experiment was performed. Oral MEKi gavages were administered 6 h after the injection of *TGFB1* mRNA, and tumors were collected 24 h later. The injected mRNA was codon-optimized, meaning single base modifications were made while maintaining the correct amino acid sequence [52]. This optimized sequence allowed for the design of qPCR primer pairs that specifically detect this sequence without recognizing the endogenously expressed mRNA. Using this approach, a substantial increase of TGFβ1 expression was observed following the injection compared to control (Fig. 7D), resulting in a trend of increased BCL2L11 levels between the MEKi-only and TGFβ1 + MEKi conditions (Fig. 7E, F). The expression of the injected mRNA is presumed to remain localized at the tumor injection site, as suggested by zsGreen protein expression confined to the injection area in a tumor injected with zsGreen mRNA. (Supplementary Fig. 4B, C). Over the course of 9 days, tumor sizes were monitored during the treatment period (Fig. 7G). MEKi-treated tumors exhibited significantly reduced growth compared to untreated (DMSO) or *TGFβ1*-treated animals. Notably, the combined treatment of MEKi and *TGFB1* mRNA further decreased tumor growth significantly compared to MEKi-only treated tumors. Efficacy of MEKi applied in vivo was confirmed by downregulated levels of pERK (Supplementary Fig. 4D). These results not only validate the in vitro findings of increased apoptosis in MEKi + TGFβ1 treated melanoma cells but also highlight the efficacy of local mRNA administration as a therapeutic approach.

## Discussion

The context-specific nature of TGFβ signaling activity has been observed across various biological processes [53]. One TGFβ signaling output is induction of apoptosis, as has been shown in various cell types, including NCSCs during embryonic development [54–58]. In cancer, TGFβ signaling has been associated with distinct steps in disease progression [9] and therapy responses, such as notably resistance formation to targeted therapy in melanoma [18, 19, 59]. Building upon the concept that tumors can co-opt embryonic programs [25], our current findings demonstrate that, akin to NCSCs, TGFβ signaling has the capacity to induce apoptosis in melanoma in a context-dependent manner. Specifically, upon trametinib-mediated inhibition of the MAPK pathway and simultaneous activation of the TGFβ pathway, downstream signaling of TGFβ shifts from enhancing invasiveness to triggering apoptosis. This effect was observed in *NRAS*- and *BRAF*-mutant, as well as in *BRAF/NRAS*- wildtype melanoma cells, indicating that the pro-apoptotic activity of TGFβ is independent of the oncogenic driver mutation. Likewise, apoptosis can be triggered by combinatorial TGFβ1 + MEKi treatment in MEKi-sensitive as well as MEKi-resistant human melanoma cells. This, together with the finding that intra-tumoral injection of synthetic *TGFB1* mRNA sensitizes human melanoma to targeted therapy in vivo, demonstrates the potential therapeutic relevance of our study.

Earlier research demonstrated that TGFβ ligands activate the SMAD2/3 pathway [60, 61] but can also engage the SMAD1/5 pathway [33, 34], with processes like EMT requiring signals from both [62]. Apoptosis has been linked to activation of the SMAD2/3 axis [63, 64], but recent findings indicate that the SMAD1 pathway can also trigger apoptosis in certain cell types [65]. However, in our study, TGFβ1 only triggered SMAD2 phosphorylation and did not affect phosphorylation of SMAD1 or SMAD5, regardless of MAPK pathway inhibition. Consequently, the transition from promoting invasiveness to inducing apoptosis in melanoma cells is likely unrelated to the phosphorylation of SMAD1/5.

Combining RNAseq with SMAD4 CUT&RUN sequencing, we reveal a novel apoptosis gene signature, which is specific to the double treatment condition and conserved across different melanoma cell lines. Among the signature genes, we validated the contribution of *UBE4B*, *BAP1* and *BCL2L11* to the apoptotic effect. Although the ubiquitin conjugation factor *UBE4B* has been linked to p53 degradation in various cancers [66–68], it is also a tumor suppressor in neuroblastoma [69, 70]. A recent studies showed that UBE4B induces apoptosis by polyubiquitinating DNA repair and anti-apoptotic proteins [71]. In addition, BAP1, a ubiquitin hydrolase, was also able to partially mediate TGFβ1 + MEKi-induced apoptosis. While BAP1’s role as a tumor suppressor is well-established in breast cancer [72], malignant mesothelioma [43, 73], and uveal melanoma [74, 75], its involvement in targeted therapy-induced apoptosis is novel. BAP1 has also been shown to release BAX and reduce anti-apoptotic BCL2 proteins [42], synergizing with BCL2L11. The finding that downregulation of UBE4B or BAP1 in BCL2L11 knockout cell lines did not further enhance the cell death rescue effect, and that mRNA-mediated upregulation of UBE4B and BAP, unlike BCL2L11, failed to induce apoptosis, strengthens the hypothesis that BCL2L11, BAP1 and UBE4B operate via the same mechanism, with BCL2L11 being the key element of this process.

*BCL2L11*, a central mediator of apoptosis in various cell types, is known to be upregulated through the SMAD2/3 pathway upon activation by TGFβ [2–4, 76]. However, our study found that *BCL2L11* expression did not increase through stimulation with TGFβ1 alone. Interestingly, MAPK pathway activation can reduce BCL2L11 levels through phosphorylation and subsequent degradation and thereby prevent apoptosis [77–79]. This process appears to be reverted upon MAPK pathway inhibition in the presence of TGFβ1, resulting in elevated BCL2L11 protein levels and, consequently, apoptosis induction in melanoma. Similarly, TGFβ-induced Dual Specificity Protein Phosphatase 4 (*DUSP4*, also known as *MKP2*) expression inhibits ERK activity in hepatocytes and prevents BCL2L11 degradation, thus promoting apoptosis [80]. However, although UBE4B, BAP1 and BCL2L11 contribute to apoptosis specifically in the double treatment context, it is conceivable that additional genes encoded in the TGFβ1 + MEKi-specific apoptosis signature are involved and that their concomitant upregulation would synergistically enhance the apoptotic effect.

While targeted therapies have significantly improved the treatment of melanoma patients in recent years, the problem of resistance formation against those therapies (even when applied in combination) remains [81, 82]. Hence, improvement of therapeutic options is clearly needed. With the observed effect of boosted apoptosis in the combination treatment with TGFβ1 + MEKi, we create a context, in which the outcome of TGFβ signaling is drastically rewired from increasing invasiveness to promoting apoptosis, even in MEKi-resistant melanoma cells. With the local administration of *TGFβ1* mRNA through intra-tumoral injections, we provide a proof-of-principle that mRNA injection could be used in clinical applications to treat melanoma. It is conceivable to use mRNA of the identified apoptosis signature genes instead of *TGFβ1* mRNA. In addition, strategies could aim to stimulate activation and tumor infiltration of immune cells which are known to produce or activate TGFβ, such as monocytes or macrophages [83], in conjunction with concurrent targeted therapy. Alternatively, the application of radiation therapy, which is known to induce TGFβ signaling [84, 85], could be an approach for locally restricted TGFβ activation and, thus, represent a potential strategy for a beneficial combinatorial therapy.

## Material and methods

### Cell lines

In this work, patient-derived human melanoma cell lines were used which were established and characterized by the URPP Live Tumor Cell Biobank, University of Zürich, including the following: M010817, M170117, M161201, M130903, MM150543, M130830, MM140325, M100916 and MM170522 (BASEC: ID.PB.2018_00194). All human melanoma cell lines were cultured in RPMI 1640 (42401018, Thermo Fisher Scientific) supplemented with 10% Fetal Bovine Serum (16140071, Thermo Fisher Scientific), 4 mM L-Glutamine (25030, Thermo Fisher Scientific) and Penicillin-Streptomycin (15070063, Thermo Fisher Scientific); the *NRAS/BRAF* wilde-type lines MM140325, M100916 and MM170522 were additionally supplemented with 10 ng/mL SCF (300-07, Peprotech). HEK-293T cells were purchased (ATCC) and cultured in DMEM/F12 (11320033, Thermo Fisher Scientific) supplemented with 10% FBS and Penicillin-Streptomycin. All cell lines were cultured at 37 °C, 5% CO2 and 95% relative humidity.

### Determination of MEKi (trametinib) IC50 values by MTT assay

Human melanoma cells were plated in 96-well plates at a density of 10.000 cells per well and allowed to attach overnight. trametinib (A-1258, Active Biochemicals) was dissolved in DMSO (Sigma Aldrich, D4540) to prepare stock solutions of 50 µM. Serial dilutions of trametinib (16 pM–250 nM) were prepared in complete culture medium and final DMSO concentration in each well did not exceed 0.5%. Cells were treated with different concentrations of trametinib for 72 h. Control wells were treated with culture medium and DMSO only. After treatment, MTT assay was performed using the MTT assay Kit (ab211091, Abcam) according to the manufacturers protocol. Absorbance was measured at 590 nm using a microplate reader (Cytation 5, Biotek). Concentration-response curves were plotted using GraphPad Prism version 9.5.1 for Windows (GraphPad Software, San Diego, California USA). Data were expressed as mean ± standard error of the mean (SEM) on a log scale and non-linear curve fit was performed. IC50 values (half maximal inhibitory concentration) of trametinib were determined for each cell line.

### In vitro treatment + apoptosis assay

Human melanoma cells were seeded in a tissue culture treated 96-well plate in complete culture medium. After 24 h, trametinib (A-1258, Active Biochemicals) and TGFβ1 (7754-BH-005/CF, R&D Systems) was applied in fresh complete culture medium. Where indicated, ZVAD (HY-16658B, MedChemExpress) was additional added. Apoptosis was assessed after 72 h of treatment by staining with Annexin V coupled fluorophores (640941, Biolegend) and (if indicated) Propidium iodide (PI) solution (421301, Biolegend) and subsequent analysis by fluorescence-activated cell sorting.

### Live cell counting using the GE INCell Analyzer 2500 HS system

Cells were seeded in complete culture medium at 10’000 cells per well in a black flat-bottom, cell culture, µCLEAR® microplate (655090, Greiner). After settling, cells were directly incubated in presence of 10 nM trametinib (A-1258, Active Biochemicals), 10 ng/mL TGFβ1 (7754-BH-005/CF, R&D Systems), 0.25 µM Sytox Green (S7020, ThermoFisher Scientific) and 32 nM Hoechst 33342 (62249, ThermoFisher Scientific). Cells were assessed for viability by acquiring both, green and blue fluorescence images at 3 h intervals at 10x magnification over 48 h using the INCell Analyzer 2500HS (GE Healthcare). Imaging was performed with equipment maintained by the Center for Microscopy and Image Analysis, University of Zurich. Sytox green and Hoechst fluorescence were measured and analyzed using the Fiji software. Number of Sytox green and Hoechst positive cells were evaluated by using a semiautomatic plug-in designed on Fiji. For every image, the channels were separated, and a particle analysis was performed on both channels. Manual threshold was set with “RenyiEntropy dark” and used to create a mask identifying objects (size exclusion was set from 10–400 pixels), followed by counting of particles. The numbers of sytox green positive cells per image were normalized over the number of Hoechst positive cells per image.

### Protein isolation and western blotting

Protein from cells or tissue was isolated using RIPA buffer (89900, Thermo Fisher Scientific) supplemented with Halt Protease and Phosphatase Inhibitor Cocktail (78440, Thermo Fisher Scientific). Protein lysates were homogenized by brief sonication. Protein quantification was done by a bicinchoninic acid assay (BCA assay) according to the manufacturer’s instructions (23225, Thermo Fisher Scientific). Denaturation of protein samples was done in Laemmli buffer (1610747, Bio-Rad) containing 10% 2-mercaptoethanol (M6250, Sigma-Aldrich) for 5 min at 95 °C. Sodium-dodecylsulfate polyacrylamide gelelectrophoresis (SDS-PAGE) was carried out on 4-20% Mini-PROTEAN TGX gels (456-1094, Bio-Rad). Transfer to nitrocellulose membranes (1704158, BioRad) was done using the Trans-Blot Turbo Transfer System (Bio-Rad). After blocking the membrane with Odyssey blocking buffer (927-40000, LI-COR Biosciences) for 1 h at room temperature, primary antibodies (see Supplementary Table) were applied in Odyssey blocking buffer in indicated dilutions overnight at 4°C. Membranes were washed in PBS containing 0.05% TWEEN 20 (P1379, Sigma-Aldrich) and 0.05% N3Na (71289, Sigma-Aldrich), before secondary antibodies (see Supplementary Table) were applied in Odyssey blocking buffer for 1 h in indicated dilutions. Membranes were washed again and visualized and quantified using an Odyssey imaging system (LI-COR Biosciences).

### TGFβ1 ELISA

Supernatant of human melanoma cells was collected 48 h after transfection using different amounts (2/20/200 ng) of zsGreen or 223/225 cys to ser mutated *TGFB1* mRNA. Concentration of TGFβ1 ligand was determined using the Human TGF-beta 1 DuoSet ELISA kit (DY240, R&D Systems) and DuoSet ELISA Ancillary Reagent Kit 1 (DY007B, R&D Systems) according to the manufacturers protocol.

### Bulk RNA sequencing

RNA isolation was performed using the NucleoSpin RNA Mini kit (740955, Macherey-Nagel) according to the manufacturer’s protocol. Quality control, library preparation and sequencing were performed at the Functional Genomics Center Zürich (FGCZ). RNA integrity numbers (RINs) were assessed on a TapeStation (Agilent). Library preparation was done using the TruSeq RNA Library Prep Kit v2 (RS-122-2001/RS-122-2002, Illumina). Samples were sequenced by a NextSeq 550 instrument (Illumina) in the single read configuration for 75 bp.

### CUT&RUN low volume urea

M170117 cells were cultured in at 37 °C, 5% CO2, and 95% humidity in RPMI medium (11875093, Thermo Scientific) supplemented with 10% Calf Bovine Serum (12133 C, Sigma-Aldrich), 10 U/ml Penicillin-Streptomycin (15140122, ThermoFisher Scientific) and 4mM L-Glutamine (25030, ThermoFisher Scientific). Passages were performed with 2 mM EDTA-PBS (AM9260G, ThermoFisher Scientific). Stimulations were performed with TGFβ1 (RnD Systems 7754-BH-005, Stock: 10 µg/mL in 4 mM HCl + 0.1% BSA, 1:5000 dilution of stock), and/or MEKi (trametinib, Stock: 50 µM Active Biochemicals—A-1258, 1:1000 dilution of stock), and/or DMSO 20 hours prior to harvest for CUT&RUN. Cells were detached with 2 mM EDTA-PBS for 5 min and then centrifuged 250 g for 5 min. Cells were resuspended in 2 ml PBS and counted; 250,000 cells were taken per replicate (N = 2 per condition). CUT&RUN Low Volume Urea, library preparation and sequencing were performed as described in [86], using anti-SMAD4 (ABIN6972727, antibodies-online), anti-SMAD4 (D3R4N #46535, Cell Signaling Technology), and IgG negative control (ABIN101961, antibodies-online). Samples were sequenced pair-end to a depth of 5–15 million reads.

### CUT&RUN data analysis

Reads were trimmed with bbmap bbduk ([87], version 38.18) to remove adapter sequences, known artifacts, and poly [AT], G and C repeats. Reads were aligned to the hg38 genome with bowtie2 ([88], version 2.4.5) with the options –local –very-sensitive-local –no-unal –no-mixed -no-discordant –phred33 –dovetail -I 0 -X 500. Samtools ([89], version 1.11) suite was used to remove duplicate and incorrectly paired reads. Bedtools ([90], version 2.30.0) was used to remove reads mapped to the CUT&RUN hg38 blacklist [91] from bam files. Individual track bedgraphs were created using bedtools genomecov on pair-end mode. For visualization, bam files of biological replicates were merged using samtools and then signal per million reads tracks were created by using the --SPRM function of macs2 ([92], version 2.2.6) for each replicate pool with the options -f BAMPE --keep-dup all --SPMR and –bdg. Peaks were individually called using macs2 for each replicate against the negative control using the options -f BAMPE –keep-dup-all and -p 1e-2. Output narrowPeak files were overlapped using Intervene ([93], version 0.6.4), keeping only reproducible peak regions called in both replicates. Venn diagrams were created using Intervene. Motif analysis was done using HOMER ([94], version 4.11) findMotifsGenome to find motifs in the hg38 genome using -size given, and peak region annotation was done with HOMER annotatePeaks on default settings. Peak set gene annotation was done using GREAT ([95], version 4.0.4) with default parameters. Signal intensity plots were created using ngsplot ([96], version 2.63) with options -N 4 -GO none -SC global. Enrichr [97] was used for pathway analysis with the Bioplanet 2019 pathways.

### siRNA transfection

For temporary dowregulation of specific genes, human melanoma cell lines were transfected with small interfering RNAs (siRNA), indicated in the Supplementary Table. Transfections were performed using jetPRIME transfection reagent (101000046, Polyplus) in combination with 20 nM siRNA as recommended by the manufacturer’s protocol. A non-targeting siRNA was used as control (4390844, Thermo Fisher Scientific). Transfection was performed at cell confluences about 70% in the presence of complete culture medium. After 24 h, transfection medium was removed, and cells were subjected to further experiments.

### mRNA transfection

Human melanoma cells were cultured under standard conditions until reaching confluency. After detaching with 2 mM EDTA in PBS, cells were counted. 200 µL culture medium with 10.000 cells (for 48/72 h setup) or 40.000 cells (for 24 h setup) were combined with 10 µL transfection agent consisting of Lipofectamine MessengerMax (LMRNA001, ThermoFisher), OptiMEM (31985062, ThermoFisher) as well as the respective amount of mRNA (2/20/200 ng) and transferred to a well of a 96-well plate. The amounts of Lipofectamine MessengerMax were adjusted to the amounts of mRNA used (0.4 µL MessengerMax on 200 ng mRNA), before bringing the total volume to 10 µL using OptiMEM. In experiments where several mRNAs were combined, equal amounts were taken resulting in the total amount as indicated. After 24 h, transfection medium was removed and exchanged with fresh culture medium or treatment containing medium, or cells were directly subjected for analysis.

### cDNA generation and RT-qPCR

RNA isolation was performed using the NucleoSpin RNA Mini kit (740955, Macherey-Nagel) according to the manufacturer’s protocol. Quantification of purified RNA was performed using a NanoDrop ND-2000 Spectrophotometer (Thermo Fisher Scientific), before generating cDNA using the Maxima First Strand cDNA Synthesis Kit (K1641, Thermo Fisher Scientific) according to the manufacturer’s protocol. Quantitative real-time PCR was done on a LightCycler 480 System (Roche) using LightCycler 480 SYBR Green I Master Mix (4707516001, Roche) with primers designed for specific genes of interest (see Supplementary Table). Every experiment was performed in three technical replicates per sample. Quantification of gene expression was normalized using the housekeeping gene USF1.

### Construction of CRISPR-Cas9 BCL2L11 gene knockout lentiviral vector

Single guide (sg) sequences targeting the human BCL2L11 gene were designed using the Vienna Bioactivity CRISPR score [98]. A non-targeting sg sequence previously described in Tian et al. [99] served as control. Exact sg sequences are shown in the Supplementary Table. sgRNA oligonucleotides were cloned into the all-in-one Cas9 carrying lentiCRISPR v2 vector (#52961, Addgene), which was a gift from Feng Zhang [100]. Chemically competent One Shot Stbl3 *E. coli* (C737303, ThermoFisher Scientific) were transformed with lentiCRISPR v2 plasmid containing sg sequences targeting BCL2L11 (sg1 or sg2) or non-targeting sg control sequence. The NucleoBond Xtra Midi Plus EF Kit (740422.50, Macherey-Nagel) was utilized to perform plasmid isolation. Sanger sequencing (Microsynth, Switzerland) was conducted to validate the proper insertion of sg sequences.

### Lentiviral production and transduction

To produce lentiviruses, HEK293T cells were transfected with a lentiviral vector, carrying transcripts of interest as well as a puromycin resistance cassette together with the lentiviral packaging plasmid psPAX2 (#12260, Addgene) and lentiviral envelope plasmid pMD2.G (#12259, Addgene). Transfections were performed as previously described [101] with minor adjustments. Lentivirus containing supernatant was harvested 48 h and 72 h after transfection and filtered through a 0.22 µm filter (83.1826, Sarstedt). Human melanoma cell lines were transduced by applying lentivirus containing supernatant mixed with equal amount of complete melanoma culture medium and 8 µg/mL polybrene (sc-134220, Santa Cruz Biotechnology) for 24 h. To select for transduced cells, Puromycin (A11138-02, ThermoFisher Scientific) at a concentration of 1 µg/mL was added to the culture medium for a minimum of 10 days.

### Determination of MEKi (Binimetinib) IC50 values by MTT assay

Cell viability and IC50 values (half maximal inhibitory concentration) were determined using Resazurin reagent (Sigma-Aldrich, Cat#R7017). Briefly, melanoma cells were seeded in 96-well culture plates and allowed to adhere overnight at 37 °C and 5% CO2. The cells were treated with different concentrations (1 nm–10 µM) of Binimetinib (MEKi) ChEMBL Id: 3187723, a selective MAP2K1 inhibitor, for 72 h. On the day of the assay, the cell culture medium was exchanged with 15 µg/mL Resazurin solution (Resazurin stock solution: 0.15 mg/ml in PBS (Gibco, Cat#10010-015). Fluorescence was read using a plate reader (exc. 535 nm, em. 595 nm). Log10[Concentration] for DMSO treated cells were arbitrary set at −5 for calculation of IC50 values using GraphPad Prism software. IC50 values for cell lines extremely resistant to MEKi could not be calculated, as flat curves were obtained.

### Transcriptomic analysis of cell lines based on response to MEKi

A filtered list of cell lines was established based on the existence of matching transcriptomic data collected at baseline from a cohort of 168 samples (USZ Dermatology Biobank) and on the absence of prior targeted therapy in the associated clinical data. The selected cell lines were then ranked by IC50 values and categorized as “Resistant” (20 highest IC50 and 3 of which IC50 = NA) or as “Sensitive” (20 lowest IC50). Average gene expression was then compared between these two groups. Genes were considered significantly changed if the fold change expression between the two groups was lower than −0.3 or bigger than 0.3 with an associated *p*-value lower than 0.05.

### bulk RNAseq data analysis of in vitro treated human melanoma cell lines

FASTQ files were aligned to the human genome annotation release 105 retrieved from Ensembl database using STAR algorithm (version 2.7.10a [102];). The output count matrices were imported in R (version 4.0.2). Analysis of the DEGs was performed with DESeq2 (version 1.21.1 [103];) with the design formula ~cell_line + treatment + cell_line:treatment. The DEGs between treatments were computed using DMSO as the reference. DEGs with a *p* adjusted value < 0.05 were considered significantly changing.

AXL, MITF and Tirosh-resistance signatures were obtained from the supplementary tables provided in Tirosh et al. [39].

Heatmap representations were made with the ComplexHeatmap package (version 2.4.3) using as input normalized counts unless otherwise indicated. For the heatmaps of AXL, MITF and Tirosh-resistance programs, the lists of DEGs between each treatment and DMSO (*p* adjusted < 0.05) were combined and then intersected with the set of genes included in each program. The values included in the input matrix were *z*-score normalized per row and genes were clustered using hierarchical clustering with euclidean distance.

In the case of treatment specific signatures for the cell lines M130830 and M170117, the lists of DEGs between each treatment and DMSO (*p* adjusted < 0.05) were combined and used for the heatmap representation. Genes were clustered using hierarchical clustering with euclidean distance and the rows were split in 5 or 7 groups for the M130830 and M170117, respectively, based on the dendrogram. Sets of genes included in groups 1 (M130830) and 7 (M170117), showing a higher expression in the double treated samples, were used for further intersection with the CUT&RUN results.

For the heatmaps showing individual representative genes, the rlog transformed values (generated with DESeq2) instead of the normalized counts were used. In the case of cell lines M130830 and M170117 the mean value for each gene was computed from the 3 replicates. The remaining cell lines, with only one replicate, were included with representative purposes. A *z*-score normalization was performed for each row (i.e a normalization of the values between treatments in a cell line-wise manner).

Gene set enrichment analyses for the AXL, MITF, and Tirosh-resistance signatures were performed with ClusterProfiler (version 3.16.1 [104]), using as input the sorted Log2FoldChange values of the DEGs (*p* adjusted value < 0.05) between the respective treatment versus DMSO. Gene set enrichment plots were generated using a modified version of the function gseaplot2 from the enrichplot package (version 1.8.1).

Treatment-specific gene sets were derived independently for each cell line (M130830 and M170117). Genes included in the sets consisted of DEGs (treatment vs DMSO) with a *p* adjusted value < 0.05 and a Log2FoldChange > 1. Briefly, the lists of selected DEGs for each treatment per cell line were intersected, the per treatment shared sets were combined in a binary matrix, and the genes uniquely found in individual treatments were retrieved as treatment-specific signatures. The derived signatures were then used for scoring the cells from the Rambow scRNAseq dataset as detailed below.

The Rambow scRNAseq dataset was obtained from the GEO database using the accession number: GSE116237 [40]. The count matrix was imported into R and processed with Seurat package (version 4.0.1). Cell annotations for the distinct cell states (pigmented, SMC, NCSC and invasive) were provided by the authors of the original paper and were assigned as cell identities for the analysis of cell signatures. Computation of the treatment-specific signature scores on the Rambow dataset was carried out using the AddModuleScore function from Seurat, and the results were displayed as violin plots generated with the VlnPlot function.

Verfaillie proliferative and invasive signatures were derived from [105] Supplementary Data 1 file. Hoek invasive (TGFβ-like signal) and proliferative (neural crest) signatures were retrieved from [106] Supplementary Data 2B.

For the analysis of apoptotic related gene sets, GO:0006915—“Apoptotic processes” were subtracted by GO:0043066—“Negative regulation of apoptosis”. These sets were retrieved from the Mouse Genome Database (https://www.informatics.jax.org/). Mouse gene sets were translated to the human orthologs with the biomaRT package (version 2.44.4). The resulting gene list of putative pro-apoptosis genes was used as input to create intersections with RNAseq and CUT&RUN data. Similarly, the pro-invasiveness gene signature was defined by combining the AXL-program [39], the Hoek invasive (TGFβ-like signal) program [106] and Verfaillie invasive signature [105].

### In vivo xenograft experiments

Patient-derived melanoma cells (M010817) were subcutaneously injected into immunocompromised athymic nude-Foxn1^nu/nu^ mice (Envigo), 5 × 10^5^ cells per animal. Tumor growth was monitored by measurements of width (*w*) and length (*L*) of the tumors and the volume was calculated using the following formula: (*W*/2 × *W*/2 × *L*/2) × 3/4 × *π.* At tumor sizes of 300 mm^3^, intratumoral mRNA injections were applied. For the tumor growth experiments a total of 3 mRNA injections were applied every 3 days; for the validation experiment, only one mRNA injection was applied, and tumors were collected 24 h later. Each tumor was injected with 50 µL 0.8× Ringer lactate solution (3570500, Braun) containing 5 µg of 223/225 cys to ser mutated *TGFβ1* or control (fLuc) coding mRNA (5′ CleanCap® and fully substituted with 1-methyl pseudouridine, produced at the Zurich mRNA platform as previously described [107, 108]). To assess *TGFβ1* and BCL2L11 expression levels, the animals received one oral gavage of trametinib, 24 h after mRNA injection and 6 h before termination. For the tumor growth experiment, 24 h after the first injection of mRNA, daily oral gavages of 1000 µg/kg trametinib (A-1258, Active Biochemicals) or vehicle solution only (10% DMSO in PBS) were applied for a total of 9 days. Animals were euthanized at the end of the treatment and tumor tissue was collected for subsequent analyses. Maximal tumor sizes of 1000 mm^3^ were not exceeded.

### Statistical analysis

The number of biological replicates is stated in the figure legends as ‘*n*’ and refers to the number of patient samples analyzed, or number of individual cell line experiments performed in this study. Unpaired Student’s *t*-test, Mann-Whitney test, analysis of variance (ANOVA), and Tukey’s (posthoc) test were performed using GraphPad Prism version 9.5.1 for Windows (GraphPad Software, San Diego, California USA) as indicated. Statistically significant differences are represented as: ns, not significant; *, *P* < 0.05; **, *P* < 0.01; ***, *P* < 0.001 and ****, *P* < 0.0001. The statistical details can also be found in the corresponding figure legends.

## Supplementary information

Supplementary Figure 1

Supplementary Figure 2

Supplementary Figure 3

Supplementary Figure 4

Supplementary figure legends

Material table

Full size western blots

## Data Availability

Newly generated bulk RNAseq datasets reported in this paper have been deposited in NCBI’s Gene Expression Omnibus and are accessible through GEO series accession number GSE262012. Scripts used for the analysis of the transcriptomics data are available at: https://github.com/adsalas/tgfb_melanoma. CUT&RUN datasets included in this paper have been deposited in the ArrayExpress database and are accessible under the accession number E-MTAB-13951. Scripts used for the analysis of the CUT&RUN data are available at: https://github.com/annanordin/CUTnRUN_SMAD4_M170117.
