# Supplementary figures and images for: TGFβ signaling sensitizes MEKi-resistant human melanoma to targeted therapy-induced apoptosis
Source: Cell Death Dis. 2024 Dec 21;15(12):925. doi: 10.1038/s41419-024-07305-1 (PMC11663225; doi:10.1038/s41419-024-07305-1)

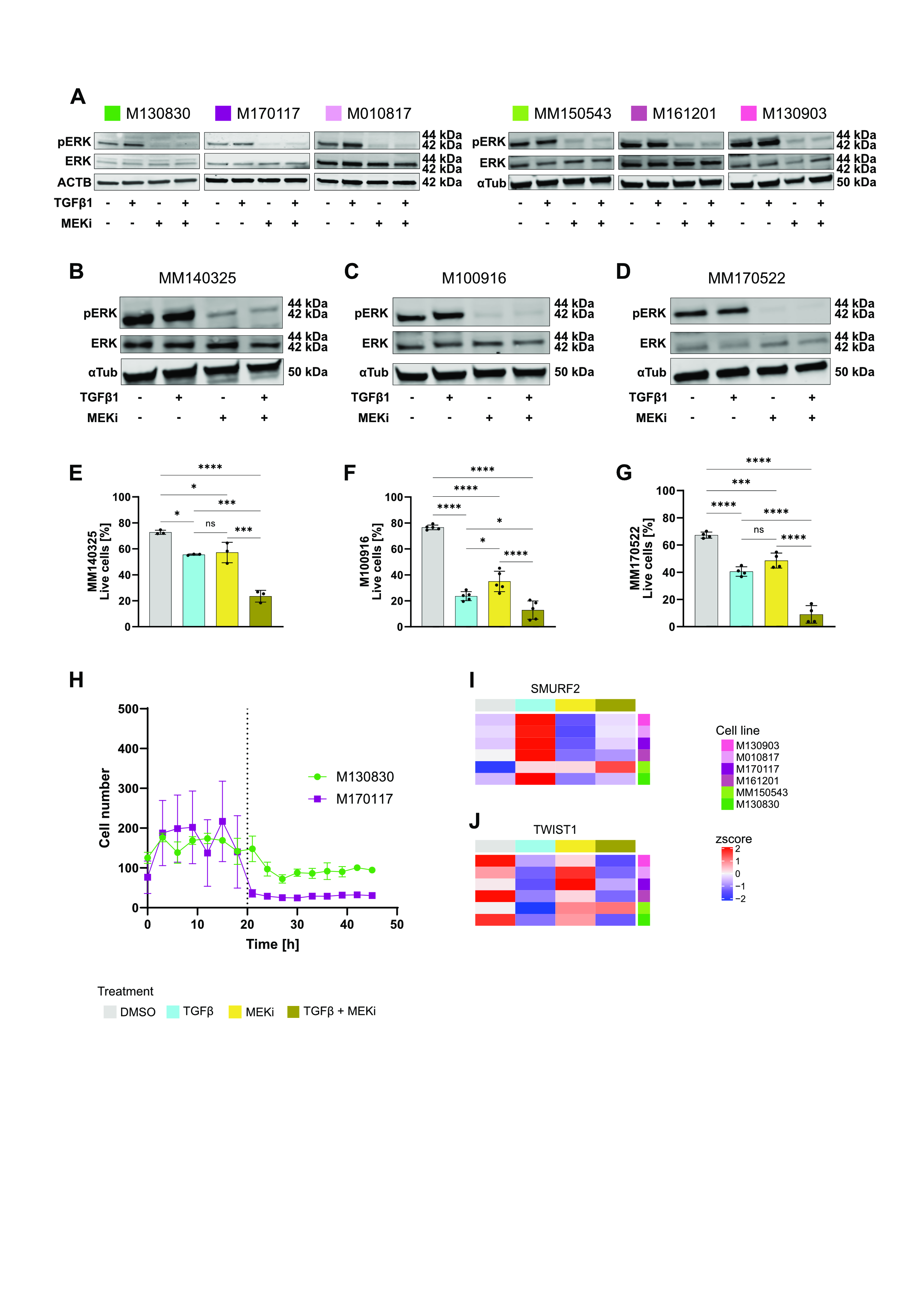

Supplement: Supplementary file 1 — Supplementary Figure 1 [file 41419_2024_7305_MOESM1_ESM.tif]

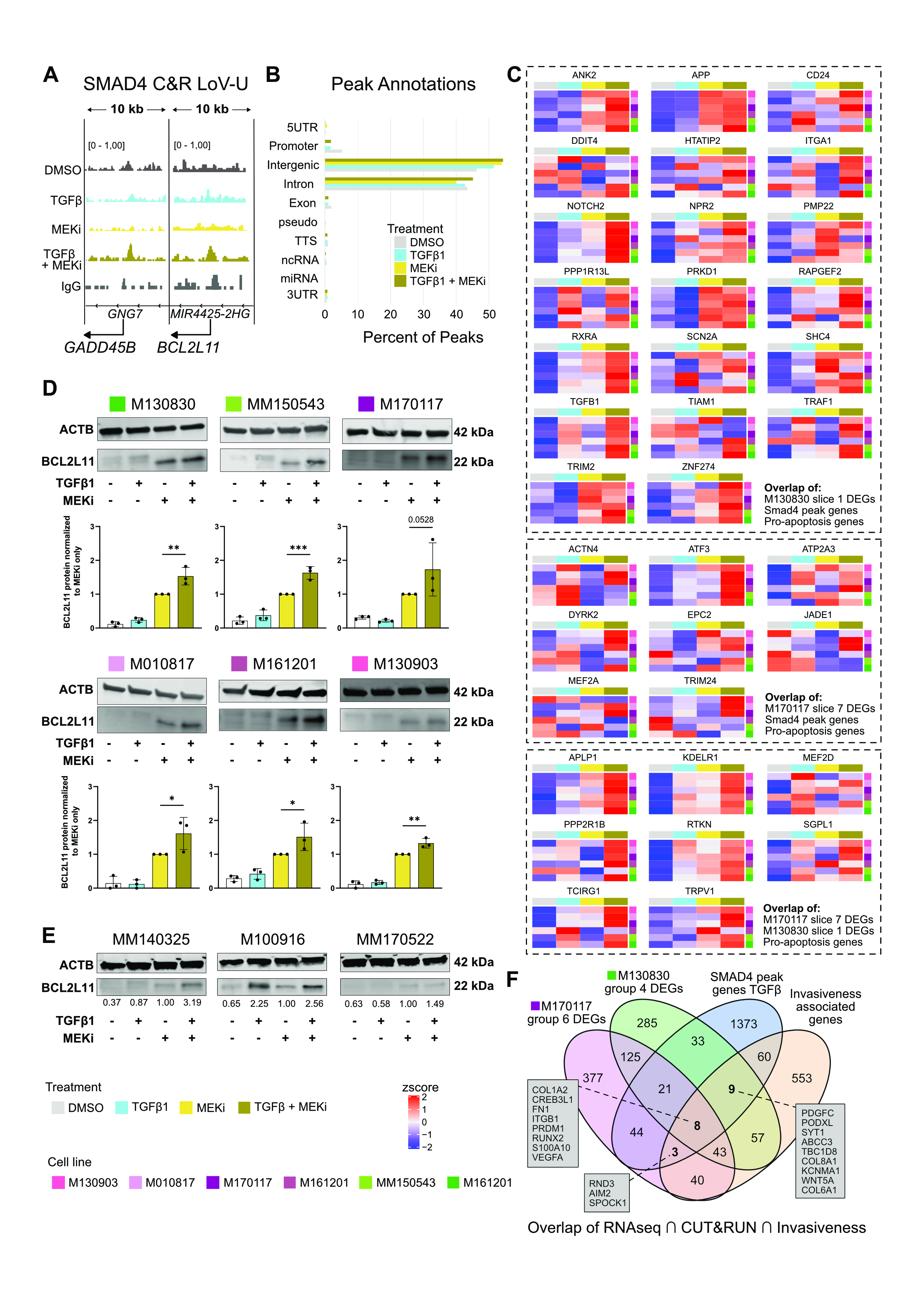

Supplement: Supplementary file 2 — Supplementary Figure 2 [file 41419_2024_7305_MOESM2_ESM.tif]

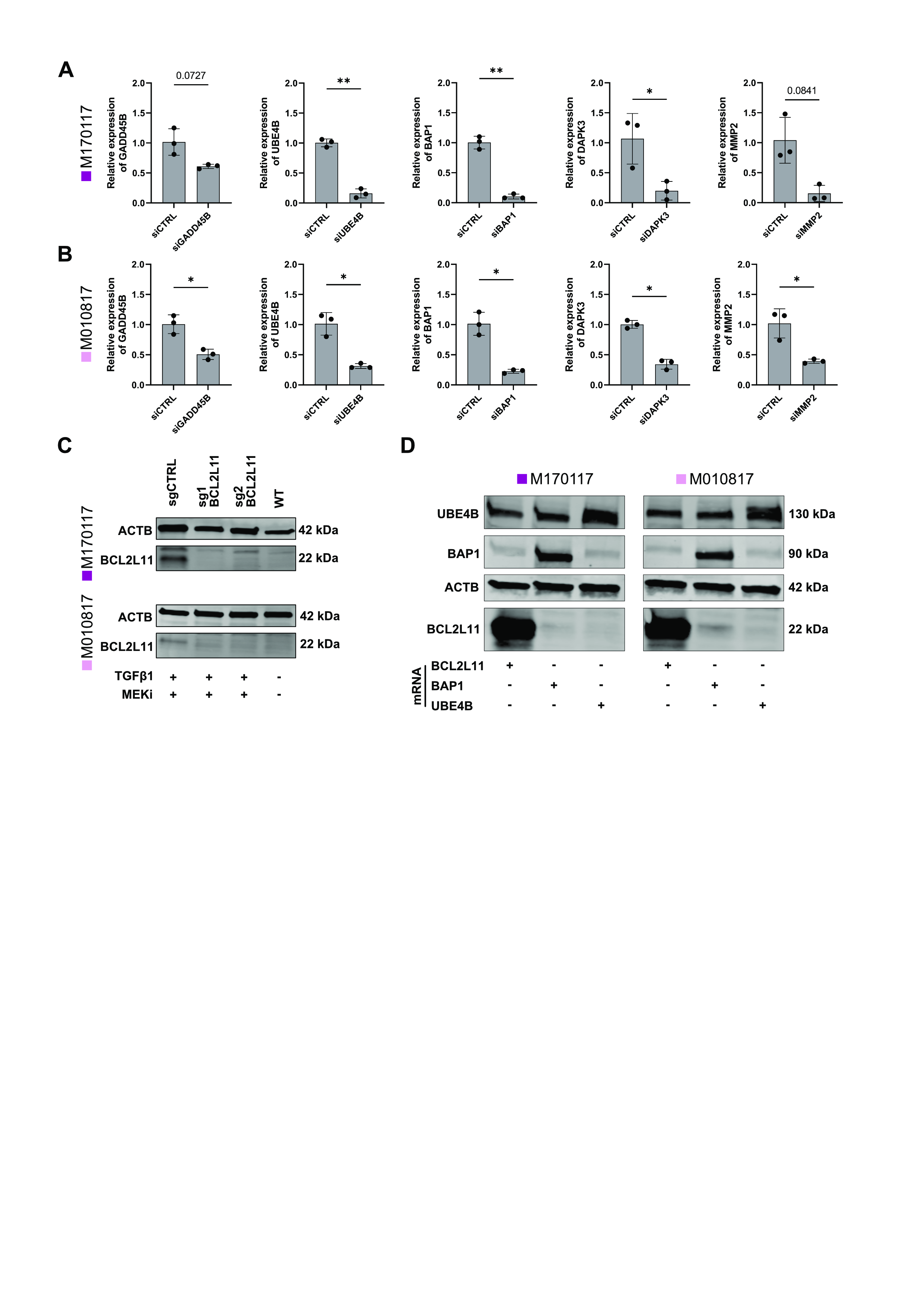

Supplement: Supplementary file 3 — Supplementary Figure 3 [file 41419_2024_7305_MOESM3_ESM.tif]

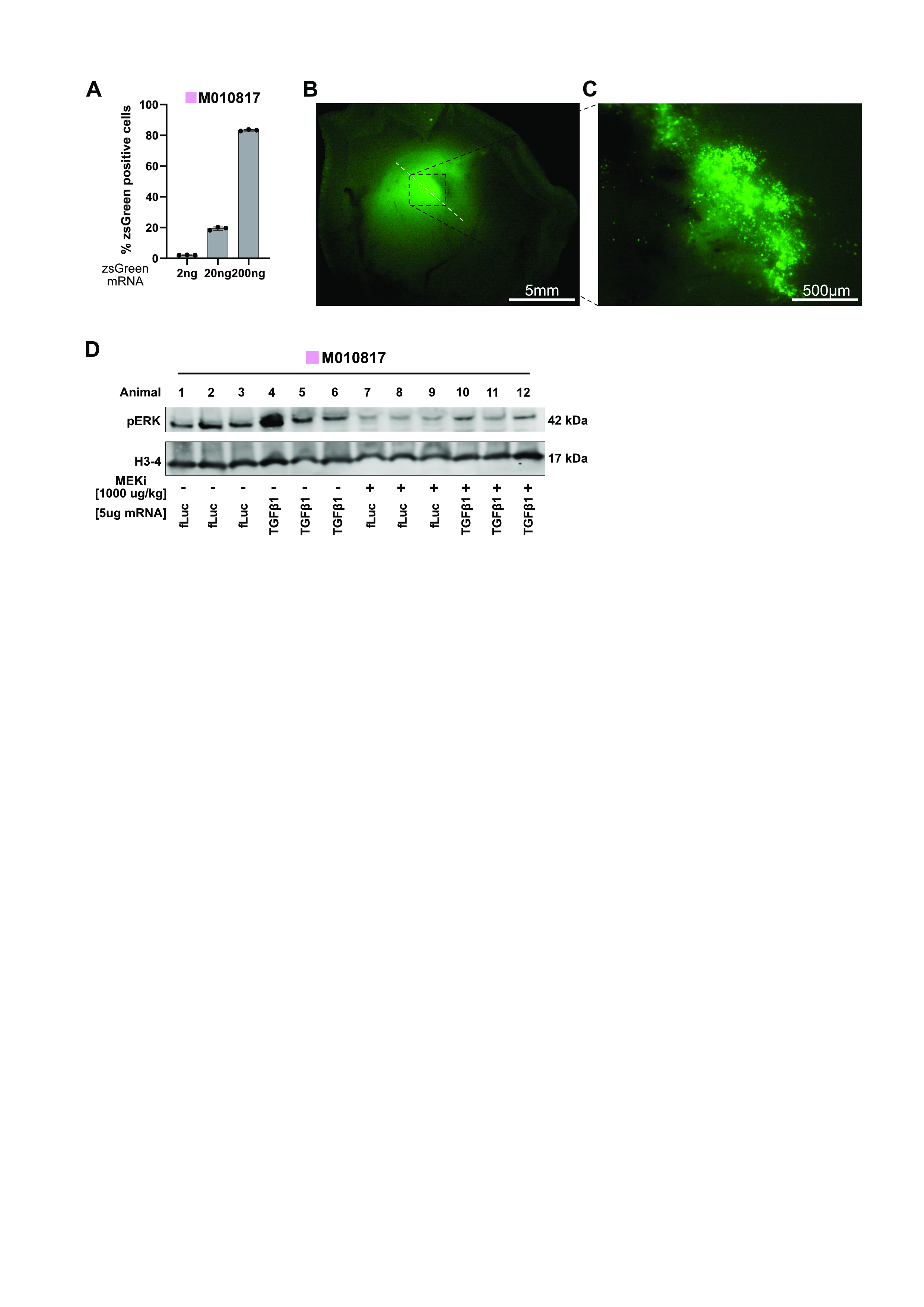

Supplement: Supplementary file 4 — Supplementary Figure 4 [file 41419_2024_7305_MOESM4_ESM.tif]
