# Supplementary material for: TGFβ signaling sensitizes MEKi-resistant human melanoma to targeted therapy-induced apoptosis
Source: Cell Death Dis. 2024 Dec 21;15(12):925. doi: 10.1038/s41419-024-07305-1 (PMC11663225; doi:10.1038/s41419-024-07305-1)
Supplement: Supplementary file 5 — Supplementary figure legends [file 41419_2024_7305_MOESM5_ESM.docx]

**Supplementary Figure 1**

(**A**) Western blots for pERK, total ERK and β-Actin (ACTB) or α-Tubulin (αTub) from whole cell lysates of two sensitive and four resistant melanoma cell lines treated for 24h with combinations of 10 ng/mL TGFβ1 and 10 nM MEKi. (**B - D**) Western blots for pERK, total ERK and α-Tubulin (αTub) from whole cell lysates of the three BRAF/NRAS wild-type melanoma lines, MM140425 (B), M100916 (C), and MM170522 (D) treated with combinations of TGFβ1 (10 ng/mL) and MEKi (10 nM) for 20h as indicated. (**E - G**) Flow cytometry quantification of Annexin V and PI double-negative cells (“Live cells”) after 72h exposure to 10 ng/mL TGFβ1 and/or 10 nM trametinib (MEKi) for three BRAF/NRAS wild-type melanoma lines, MM140425 (E), M100916 (F), and MM170522 (G). (**H**) Growth curves of two human melanoma cell lines (sensitive M130830 and resistant M170117) during combination treatment of 10 ng/mL TGFβ1 and 10 nM MEKi assessed every 3h over a period of 48h. Hoechst 33342 positive cell counts are shown. (**I + J**) Heatmaps showing the expression of *SMURF2* (I) and *TWIST1* (J) from bulk RNA-seq data of six melanoma cell lines 20h after TGFβ1 / MEKi combinatorial treatment.

**Supplementary Figure 2**

(**A**) Examples of peak associated genes identified by SMAD4 CUT&RUN exclusively in the TGFβ1 + MEKi condition visualized in Integrative Genome Viewer (see also Figure 3C). (**B**) Bar graph showing DNA peak regions annotated by HOMER for the four treatment conditions. (**C**) Heatmaps of all genes derived from overlaps generated in Figure 4E, assigned to each subset showing gene expression in the four different treatment conditions DMSO, TGFβ1, MEKi and TGFβ1 + MEKi for six human melanoma cell lines. Expression values were z-score normalized per row. (**D**) Representative western blots for BCL2L11 and ACTB of six melanoma cell lines after 20h of treatment with combinations of TGFβ1 and MEKi as indicated below. BCL2L11 levels of three independent western blots were quantified and normalized to MEKi only treatment in bar graphs. P-values were calculated by ordinary one-way ANOVA and multiple comparisons for selected pairs with *p < 0.05, **p < 0.01, ***p < 0.001 and ****p < 0.0001. (**E**) Western blots for BCL2L11 and β-ACTIN (ACTB) from whole cell lysates of three BRAF/NRAS wild-type melanoma lines, MM140425, M100916, and MM170522 treated with combinations of TGFβ1 (10 ng/mL) and MEKi (10 nM) for 20h as indicated. The quantification of BCL2L11 levels is shown below, normalized to the loading control ACTB and to the MEKi only condition. (**F**) Venn diagram showing overlaps of differentially expressed genes (DEGs) in the TGFβ1 only condition for the two cell lines (M130830 group 4; M170117 group 6) as well as peak associated genes for the TGFβ1 only condition identified by SMAD4 CUT&RUN for the M170117 cell line and a pre-defined pro-invasiveness gene signature. Genes lists of each subset of interest are shown in grey boxes.

**Supplementary Figure 3**

(**A** **+** **B**) Validation of gene expression by qPCR after siRNA mediated gene knock-down, relative to control sample (siCTRL) in M170117 (A) and M010817 (B) cells. Data of three independent replicates are shown. P-values were calculated by using paired, two-tailed t-tests with *p < 0.05, **p < 0.01, ***p < 0.001 and ****p < 0.0001. (**C**) Validation of CRISPR/Cas9 mediated knock-down of BCL2L11 in M170117 and M010817 cells by western blot. (**D**) Validation of mRNA mediated upregulation of BCL2L11, BAP1, and UBE4B, 6h after transfection in M170117 and M010817 cells by western blot.

**Supplementary Figure 4**

(**A**) Validation of zsGreen expression after transfection with different amounts of zsGreen mRNA (2/20/200 ng) by flow cytometry in M010817 cells. (**B + C**) Representative fluorescence images of a xenograft melanoma tumor, 24h after injection with zsGreen mRNA. The whole tumor (B) was cut at the indicated white dashed line and a magnification image of the cut area was taken (C). (**D**) Western blot for pERK and Histone H3 (H3-4) levels in xenograft tumors upon oral MEKi (trametinib) treatment and/or intra-tumoral injections of TGFβ1 or control (fLuc) mRNA in nude mice.
