# Supplementary material for: TGFβ signaling sensitizes MEKi-resistant human melanoma to targeted therapy-induced apoptosis
Source: Cell Death Dis. 2024 Dec 21;15(12):925. doi: 10.1038/s41419-024-07305-1 (PMC11663225; doi:10.1038/s41419-024-07305-1)
Supplement: Supplementary file 6 — Material table [file 41419_2024_7305_MOESM6_ESM.docx]

**Material table:**

| **Antibodies** | | | | | | | | | | |
| --- | --- | --- | --- | --- | --- | --- | --- | --- | --- | --- |
| **Target** | | | | | **Supplier** | | **Cat. No.** | | | **Dilution** |
| **Primary antibodies for Western Blot** | | | | | | | | | | |
| β-ACTIN | | | | | Sigma-Aldrich | | A5316 | | | 1:10.000 |
| Histone H3 | | | | | Cell signaling | | 3638S | | | 1:1000 |
| α-Tubulin | | | | | Sigma Aldrich | | T6074 | | | 1:2000 |
| p44/42 MAPK (ERK1/2) | | | | | Cell signaling | | 4696 | | | 1:2.000 |
| Phospho-p44/42 MAPK (ERK1/2) | | | | | Cell signaling | | 9101 | | | 1:1.000 |
| SMAD2 | | | | | Abcam | | Ab305325 | | | 1:1000 |
| pSMAD2 | | | | | Cell signaling | | 18338 | | | 1:500 |
| SMAD1 | | | | | Abcam | | Ab53745 | | | 1:50 |
| pSMAD1/5/8 | | | | | Cell signaling | | 9516 | | | 1:500 |
| BIM (BCL2L11) | | | | | Abcam | | ab32158 | | | 1:1.000 |
| Cleaved Caspase 3 | | | | | Cell signaling | | 9661 | | | 1:1000 |
| PARP | | | | | Cell signaling | | 9542 | | | 1:1000 |
| BAP1 | | | | | Santa Cruz | | sc-28383 | | | 1:100 |
| UBE4B | | | | | Abcam | | ab126759 | | | 1:1000 |
| **Secondary antibodies for Western Blot** | | | | | | | | | | |
| IRDye® 680LT Donkey anti-Rabbit IgG | | | | | LI-COR | | 926-68023 | | | 1:6.000 |
| IRDye® 800CW Donkey anti-Mouse IgG | | | | | LI-COR | | 926-32212 | | | 1:6.000 |
| **Antibodies for CUT&RUN** | | | | | | | | | | |
| SMAD4 | | | | | antibodies-online | | ABIN6972727 | | | |
| SMAD4 | | | | | Cell signaling | | 46535 | | | |
| IgG negative control | | | | | antibodies-online | | ABIN101961 | | | |
| **Human qRT-PCR primers** | | | | | | | | | | |
| **Target gene** | **Forward sequence (5’-3’)** | | | | | **Reverse sequence (5’-3’)** | | | | |
| USF1 | TACTACCCAGGGCTCAGAGG | | | | | TCCCTGCAGTACTTCTTGTGG | | | | |
| GADD45B | GCCAGGATCGCCTCACAGTGG | | | | | GGATTTGCAGGGCGATGTCATC | | | | |
| UBE4B | CGCGTGTGAATGCAACGATGGA | | | | | GCGGATATAGCGACGGCAACTA | | | | |
| BAP1 | GAGGATGACGTGCAGAACACCA | | | | | CTCAGCCAAGACGTTGATGGTG | | | | |
| DAPK3 | AAGCAGGAGACGCTCACCAACA | | | | | CTCTGGGCAATGGTCATTCTCC | | | | |
| MMP2 | AGCGAGTGGATGCCGCCTTTAA | | | | | CATTCCAGGCATCTGCGATGAG | | | | |
| TGFB1 (codon optimized) | CCTTTGATGTCACCGGAGTT | | | | | GAACCCGTTGATGTCCACTT | | | | |
| **siRNA** | | | | | | | | | | |
| **Target gene** | | | | **Supplier** | | | **Cat. No.** | | | |
| Select Negative Control | | | | ThermoFisher Sientific | | | 4390844 | | | |
| GADD45B | | | | ThermoFisher Sientific | | | s9139 | | | |
| UBE4B | | | | ThermoFisher Sientific | | | s556 | | | |
| BAP1 | | | | ThermoFisher Sientific | | | s15822 | | | |
| DAPK3 | | | | ThermoFisher Sientific | | | s559 | | | |
| MMP2 | | | | ThermoFisher Sientific | | | s8852 | | | |
| **sgRNA sequences for CRISPR mediated gene knock-out** | | | | | | | | | | |
| **Target** | | | **sgRNA sequence + NGG** | | | | | | | |
| sgCTRL | | | GAGACGAGGACATGTGTAGC | | | | | | | |
| BCL2L11 (sg1) | | | AGTTCTGAGTGTGACCGAGAAGG | | | | | | | |
| BCL2L11 (sg2) | | | GCCCAAGAGTTGCGGCGTATTGG | | | | | | | |
| **Plasmids** | | | | | | | | | | |
| **Name** | | | | **Supplier** | | | | | **Cat. No.** | |
| lentiCRISPRv2 | | | | Addgene, Feng Zhang | | | | | 52961 | |
| psPAX2 | | | | Addgene, Didier Trono | | | | | 12260 | |
| pMD2.G | | | | Addgene, Didier Trono | | | | | 12259 | |
| **Bacteria strains** | | | | | | | | | | |
| **Name** | | | | **Supplier** | | | | | **Cat. No.** | |
| One Shot™ Stbl3™ Chemically Competent E. coli | | | | ThermoFisher Scientific | | | | | 52961 | |
| **Chemicals, peptides, and recombinant proteins** | | | | | | | | | | |
| **Name** | | | | **Supplier** | | | | **Cat. No.** | | |
| Human TGFb1 recombinant protein | | | | RnD Systems | | | | 7754-BH-005 | | |
| Trametinib | | | | Active Biochemicals | | | | A-1258 | | |
| APC Annexin V | | | | Biolegend | | | | 640941 | | |
| Propidium Iodide (PI) Solution | | | | Biolegend | | | | 421301 | | |
| Dimethyl sulfoxide (DMSO) | | | | Sigma-Aldrich | | | | D4540 | | |
| RPMI 1640 Medium | | | | ThermoFisher Scientific | | | | 42401018 | | |
| DMEM/F12 Medium | | | | ThermoFisher Scientific | | | | 11320033 | | |
| L-Glutamine | | | | ThermoFisher Scientific | | | | 25030 | | |
| Penicillin-Streptomycin | | | | ThermoFisher Scientific | | | | 15140122 | | |
| Fetal Bovine Serum (FBS) | | | | ThermoFisher Scientific | | | | 16140 | | |
| Calf Bovine Serum | | | | Sigma-Aldrich | | | | 12133C | | |
| Fetal Bovine Serum (FBS) | | | | Biowest | | | | S-181H-500 | | |
| Ethylenediaminetetraacetic acid (EDTA) | | | | ThermoFisher Scientific | | | | AM9261 | | |
| Laemmli Sample Buffer, 4x | | | | Bio-Rad | | | | 1610747 | | |
| Odyssey Blocking Buffer | | | | LI-COR Biosciences | | | | 927-40000 | | |
| RIPA Buffer | | | | ThermoFisher Scientific | | | | 89900 | | |
| Halt™ Protease and Phosphatase Inhibitor Cocktail | | | | ThermoFisher Scientific | | | | 78440 | | |
| Tris/Glycine Buffer, 10X | | | | Bio-Rad | | | | 1610734 | | |
| Tris/Glycine/SDS Buffer, 10X | | | | Bio-Rad | | | | 1610732 | | |
| TWEEN 20 | | | | Sigma-Aldrich | | | | P1379 | | |
| Sodium azide (N_3_Na) | | | | Sigma-Aldrich | | | | 71289 | | |
| Polybrene | | | | Santa Cruz Biotechnology | | | | sc-134220 | | |
| Puromycin | | | | ThermoFisher Scientific | | | | A11138-02 | | |
| SYBR Green I Master Mix | | | | Roche | | | | 4707516001 | | |
| BsmBI | | | | ThermoFisher Scientific | | | | ER0451 | | |
| Fast AP | | | | ThermoFisher Scientific | | | | EF0651 | | |
| T4 PNK | | | | New England Biolabs | | | | B0202S | | |
| T4 DNA Ligase | | | | New England Biolabs | | | | EL0011 | | |
| Hoechst 33342 | | | | ThermoFisher Scientific | | | | 62249 | | |
| Sytox Green | | | | ThermoFisher Scientific | | | | S7020 | | |
| 2-mercaptoethanol | | | | Sigma-Aldrich | | | | M6250 | | |
| EDTA-PBS | | | | ThermoFisher Scientific | | | | AM9260G | | |
| Resazurin sodium salt | | | | Sigma-Aldrich | | | | R7017 | | |
| PBS | | | | ThermoFisher Scientific | | | | 10010-015 | | |
| Ringer Lactate solution | | | | Braun | | | | 3570500 | | |
| OptiMEM | | | | ThermoFisher | | | | 31985062 | | |
| ZVAD-FMK | | | | MedChemExpress | | | | HY-16658B | | |
| Recombinant Human SCF | | | | Peprotech | | | | 300-07 | | |
| **Critical commercial assays** | | | | | | | | | | |
| **Name** | | | | **Supplier** | | | | **Cat. No.** | | |
| Pierce™ BCA Protein Assay Kit | | | | ThermoFisher Scientific | | | | 23227 | | |
| NucleoSpin RNA kit | | | | Macherey-Nagel | | | | 740955 | | |
| NucleoBond Xtra Midi Plus EF Kit | | | | Macherey-Nagel | | | | 74042250 | | |
| JetPRIME Transfection Kit | | | | Polyplus | | | | 114-15 | | |
| Lipofectamine MessengerMax | | | | ThermoFisher | | | | LMRNA001 | | |
| MTT Cell Proliferation Assay Kit | | | | Abcam | | | | Ab211091 | | |
| TruSeq RNA Library Prep Kit v2 | | | | Illumina | | | | RS-122-2001/RS-122-2002 | | |
| Maxima First Strand cDNA Synthesis Kit | | | | ThermoFisher Scientific | | | | K1641 | | |
| TGF-beta 1 DuoSet ELISA kit | | | | R&D Systems | | | | DY240 | | |
| DuoSet ELISA Ancillary Reagent Kit 1 | | | | R&D Systems | | | | DY007B | | |
| **Cell lines** | | | | | | | | | | |
| **Name** | | | | **Supplier** | | | | **Cat. No.** | | |
| Human M170117 | | | | URPP Live Tumor Cell Biobank, UZH | | | | N/A | | |
| Human M130830 | | | | URPP Live Tumor Cell Biobank, UZH | | | | N/A | | |
| Human M010817 | | | | URPP Live Tumor Cell Biobank, UZH | | | | N/A | | |
| Human MM150543 | | | | URPP Live Tumor Cell Biobank, UZH | | | | N/A | | |
| Human M161201 | | | | URPP Live Tumor Cell Biobank, UZH | | | | N/A | | |
| Human M130903 | | | | URPP Live Tumor Cell Biobank, UZH | | | | N/A | | |
| Human MM140325 | | | | URPP Live Tumor Cell Biobank, UZH | | | | N/A | | |
| Human M100916 | | | | URPP Live Tumor Cell Biobank, UZH | | | | N/A | | |
| Human MM170522 | | | | URPP Live Tumor Cell Biobank, UZH | | | | N/A | | |
| Human HEK-293T cell line | | | | ATCC | | | | CRL-3216; RRID: CVCL_0063 | | |
| **Software** | | | | | | | | | | |
| **Name** | | **Provider** | | | | **Link** | | | | |
| FlowJo (v10.6.2) | | FloJo | | | | https://www.flowjo.com/ | | | | |
| GraphPad Prism (v9.5.1 for Windows) | | GraphPad Software | | | | https://www.graphpad.com/ | | | | |
| Biorender | | Science Suite Inc. | | | | https://help.biorender.com/ | | | | |
| Affinity Designer (v.1.10.6.1665) | | Serif (Europe) Ltd | | | | https://affinity.serif.com/en-us/ | | | | |
| bbmap bbduk (v38.18) | | Joint Genome Institute | | | | https://jgi.doe.gov/ | | | | |
| bowtie2 (v.2.4.5) | | Johns Hopkins University | | | | <https://bowtie-bio.sourceforge.net/bowtie2/> | | | | |
| Samtools (v.1.11) | | Github | | | | <https://samtools.sourceforge.net/> | | | | |
| Bedtools (v.2.30.0) | | Github | | | | <https://bedtools.readthedocs.io/> | | | | |
| Intervene (v.0.6.4) | | Github | | | | <https://intervene.readthedocs.io/> | | | | |
| HOMER (v.4.11) | | University of California, San Diego | | | | <http://homer.ucsd.edu/homer/> | | | | |
| GREAT (v.4.0.4) | | Stanford University | | | | http://great.stanford.edu/public/html/ | | | | |
| Ngsplot (v.2.63) | | Github | | | | <https://github.com/shenlab-sinai/ngsplot> | | | | |
| STAR aligner (v.2.7.10a) | | Github | | | | <https://github.com/alexdobin/STAR/releases> | | | | |
| R (v.4.0.2) | | The R Foundation | | | | <https://www.R-project.org/> | | | | |
| DESeq2 (v.1.21.1) | | Bioconductor | | | | http://www.bioconductor.org/packages/release/bioc/html/DESeq2.html | | | | |
| ClusterProfiler (v.3.16.1) | | Bioconductor | | | | <http://bioconductor.org/packages/release/bioc/html/clusterProfiler.html> | | | | |
