# Supplementary material for: TGFβ signaling sensitizes MEKi-resistant human melanoma to targeted therapy-induced apoptosis
Source: Cell Death Dis. 2024 Dec 21;15(12):925. doi: 10.1038/s41419-024-07305-1 (PMC11663225; doi:10.1038/s41419-024-07305-1)

Figure 1 H

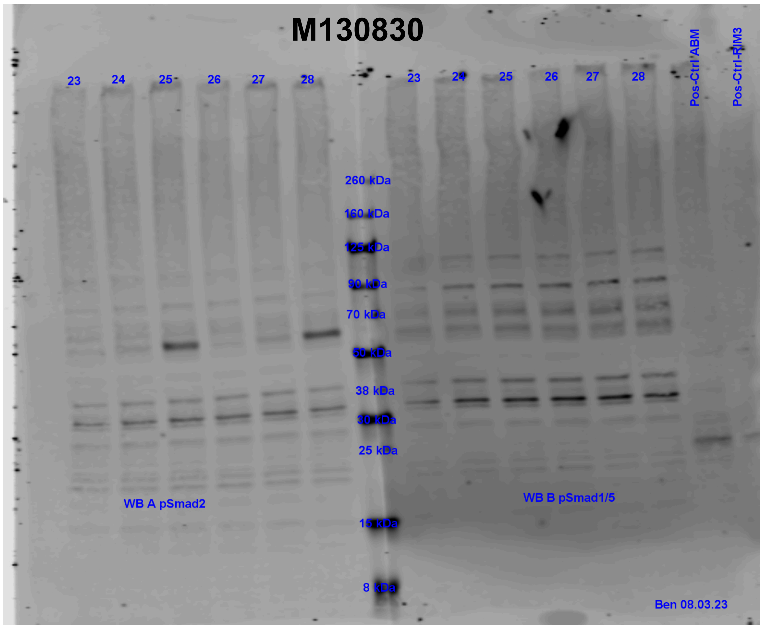
M130830
23
24
25
26
27
28
23
24
25
26
27
28
Pos-Ctrl ABM
Pos-Ctrl-RIM3
260 kDa
160 kDa
125 kDa
90 kDa
70 kDa
50 kDa
38 kDa
30 kDa
25 kDa
15 kDa
8 kDa
WB A pSmad2
WB B pSmad1/5
Ben 08.03.23

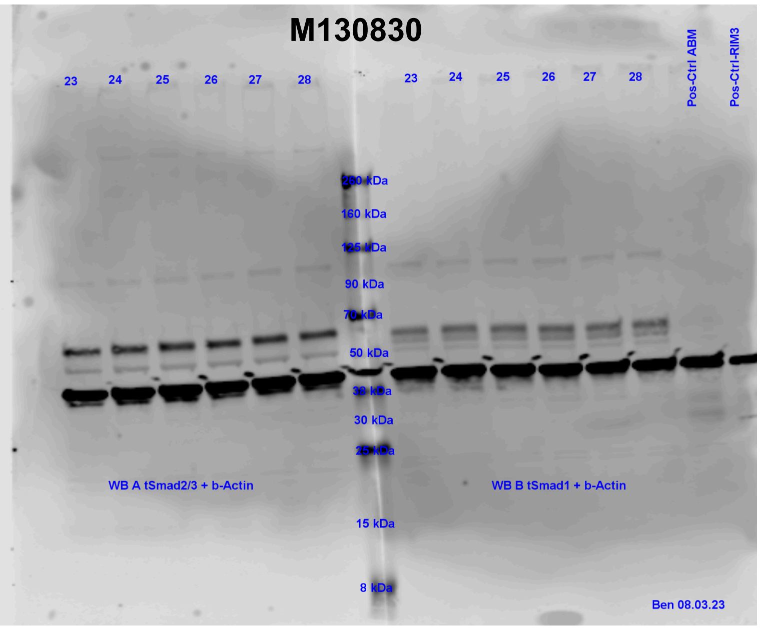
M130830
23
24
25
26
27
28
23
24
25
26
27
28
Pos-Ctrl ABM
Pos-Ctrl-RIM3
260 kDa
160 kDa
125 kDa
90 kDa
70 kDa
50 kDa
38 kDa
30 kDa
25 kDa
15 kDa
8 kDa
WB A tSmad2/3 + b-Actin
WB B tSmad1 + b-Actin
Ben 08.03.23

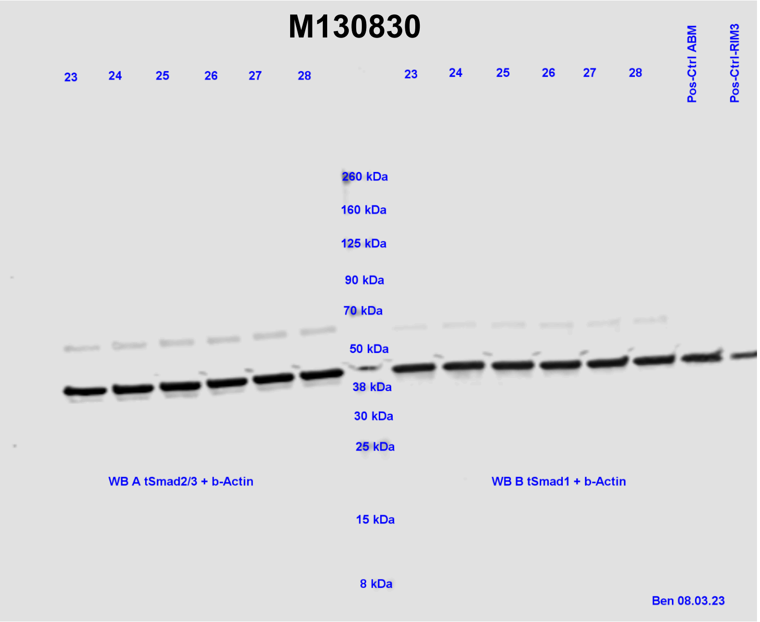
M130830
23
24
25
26
27
28
23
24
25
26
27
28
Pos-Ctrl ABM
Pos-Ctrl-RIM3
260 kDa
160 kDa
125 kDa
90 kDa
70 kDa
50 kDa
38 kDa
30 kDa
25 kDa
15 kDa
8 kDa
WB A tSmad2/3 + b-Actin
WB B tSmad1 + b-Actin
Ben 08.03.23

Figure 1 I

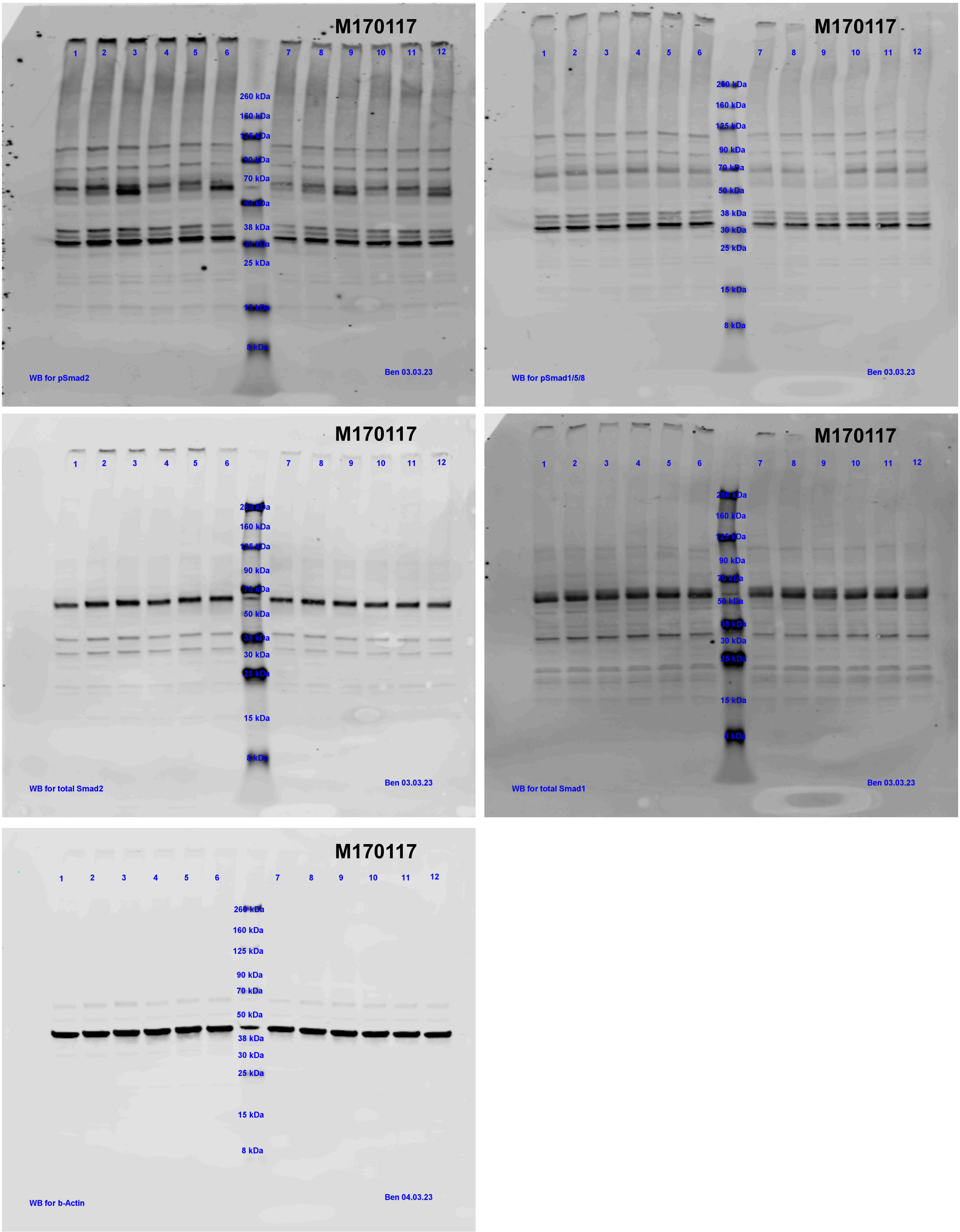
M170117
1 2 3 4 5 6 7 8 9 10 11 12
260 kDa
160 kDa
125 kDa
90 kDa
70 kDa
50 kDa
38 kDa
30 kDa
25 kDa
15 kDa
8 kDa
WB for pSmad2
Ben 03.03.23
M170117
1 2 3 4 5 6 7 8 9 10 11 12
260 kDa
160 kDa
125 kDa
90 kDa
70 kDa
50 kDa
38 kDa
30 kDa
25 kDa
15 kDa
8 kDa
WB for pSmad1/5/8
Ben 03.03.23
M170117
1 2 3 4 5 6 7 8 9 10 11 12
260 kDa
160 kDa
125 kDa
90 kDa
70 kDa
50 kDa
38 kDa
30 kDa
25 kDa
15 kDa
8 kDa
WB for total Smad2
Ben 03.03.23
M170117
1 2 3 4 5 6 7 8 9 10 11 12
260 kDa
160 kDa
125 kDa
90 kDa
70 kDa
50 kDa
38 kDa
30 kDa
25 kDa
15 kDa
8 kDa
WB for total Smad1
Ben 03.03.23
M170117
1 2 3 4 5 6 7 8 9 10 11 12
260 kDa
160 kDa
125 kDa
90 kDa
70 kDa
50 kDa
38 kDa
30 kDa
25 kDa
15 kDa
8 kDa
WB for b-Actin
Ben 04.03.23

Figure 2G

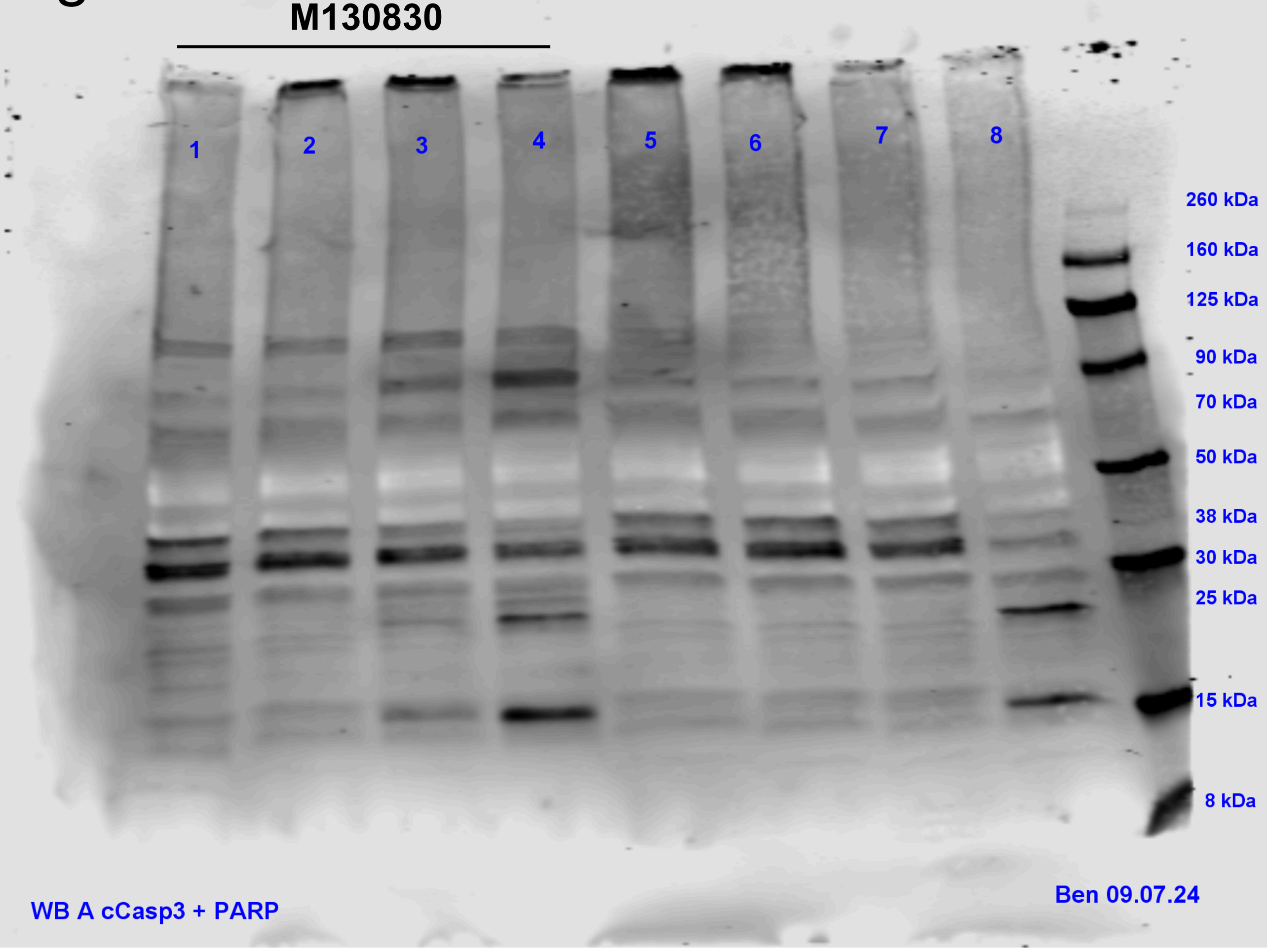
M130830
1
2
3
4
5
6
7
8
260 kDa
160 kDa
125 kDa
90 kDa
70 kDa
50 kDa
38 kDa
30 kDa
25 kDa
15 kDa
8 kDa
WB A cCasp3 + PARP
Ben 09.07.24

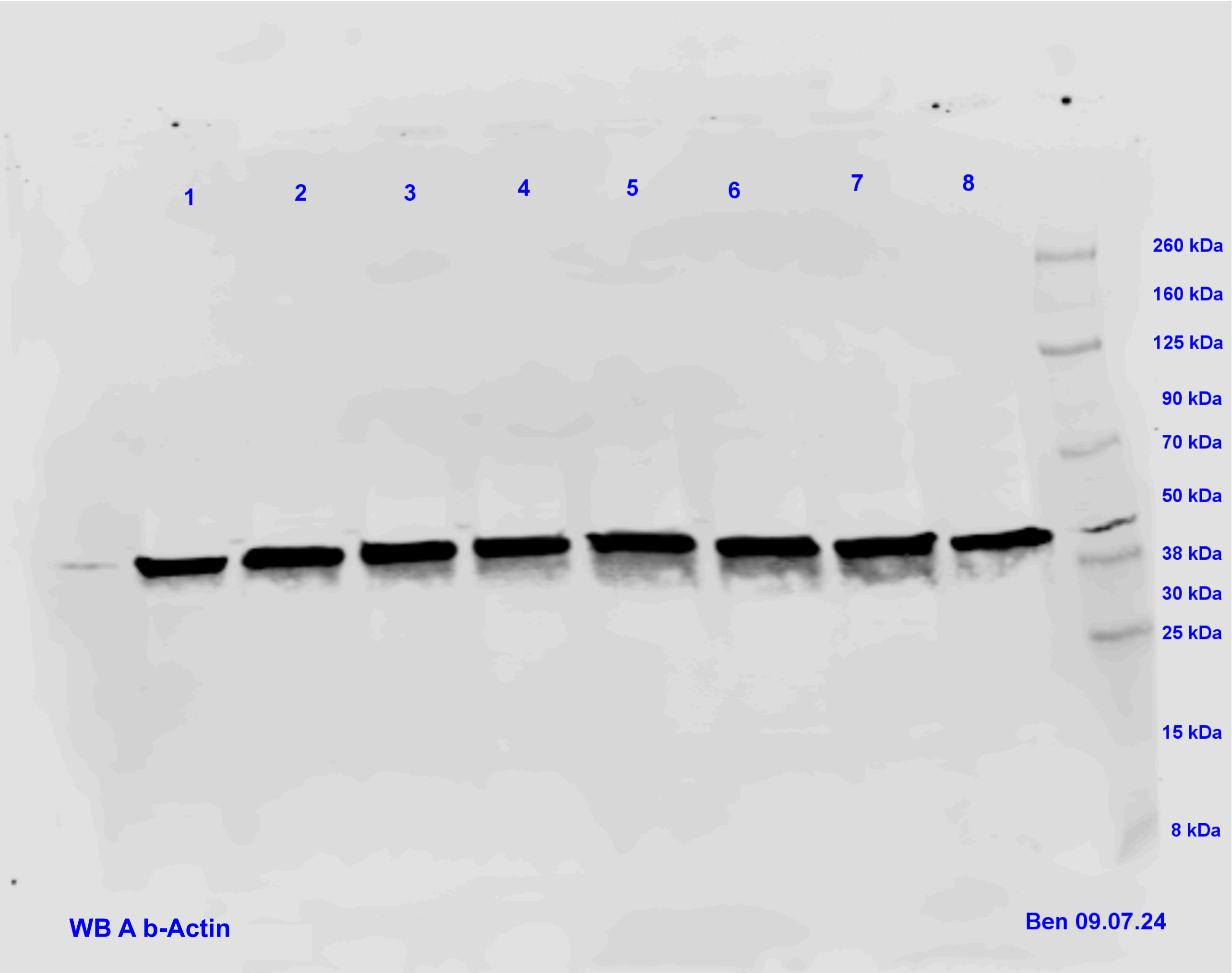
1
2
3
4
5
6
7
8
260 kDa
160 kDa
125 kDa
90 kDa
70 kDa
50 kDa
38 kDa
30 kDa
25 kDa
15 kDa
8 kDa
WB A b-Actin
Ben 09.07.24

Figure 2 H + I

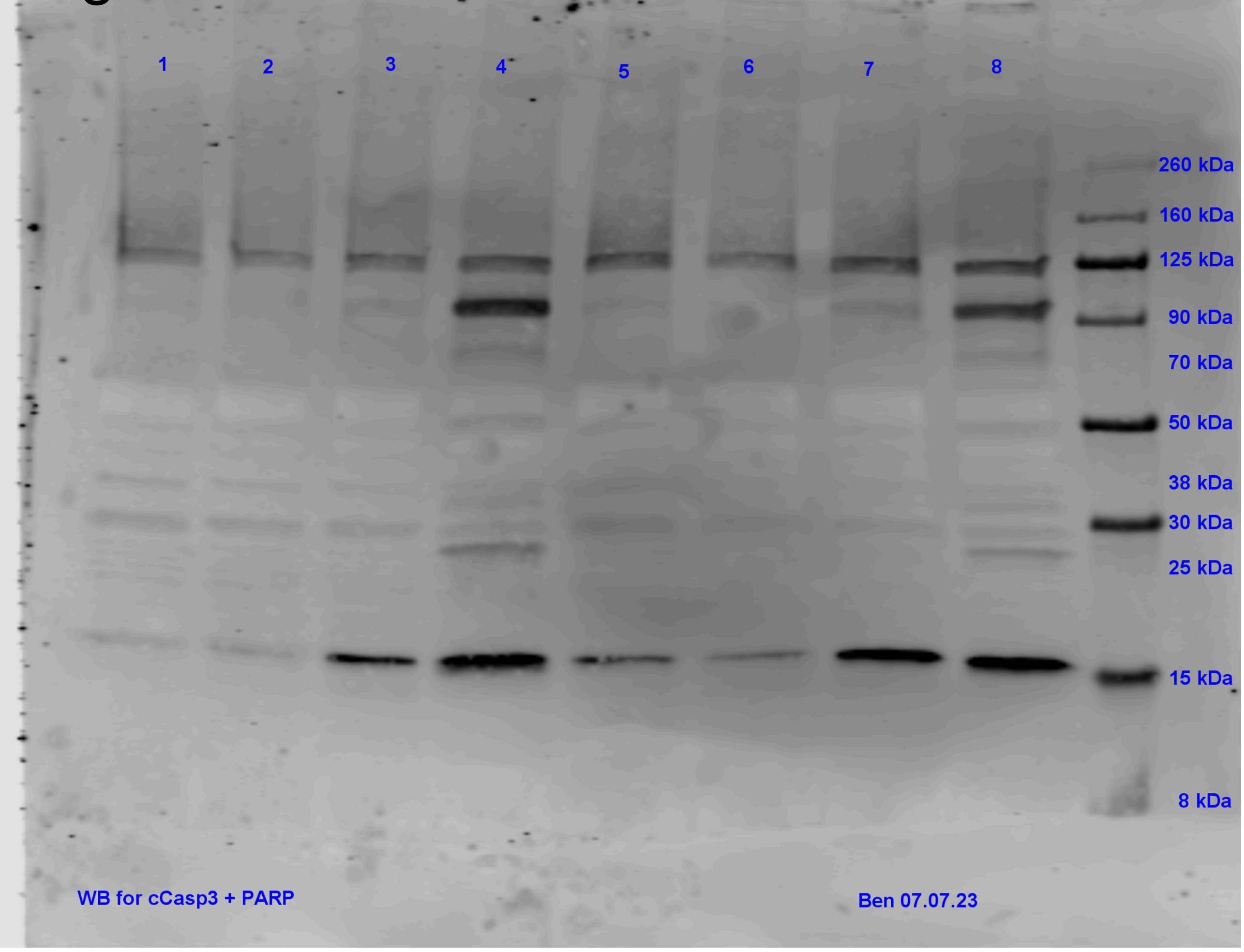

1
2
3
4
5
6
7
8
260 kDa
160 kDa
125 kDa
90 kDa
70 kDa
50 kDa
38 kDa
30 kDa
25 kDa
15 kDa
8 kDa
WB for cCasp3 + PARP
Ben 07.07.23

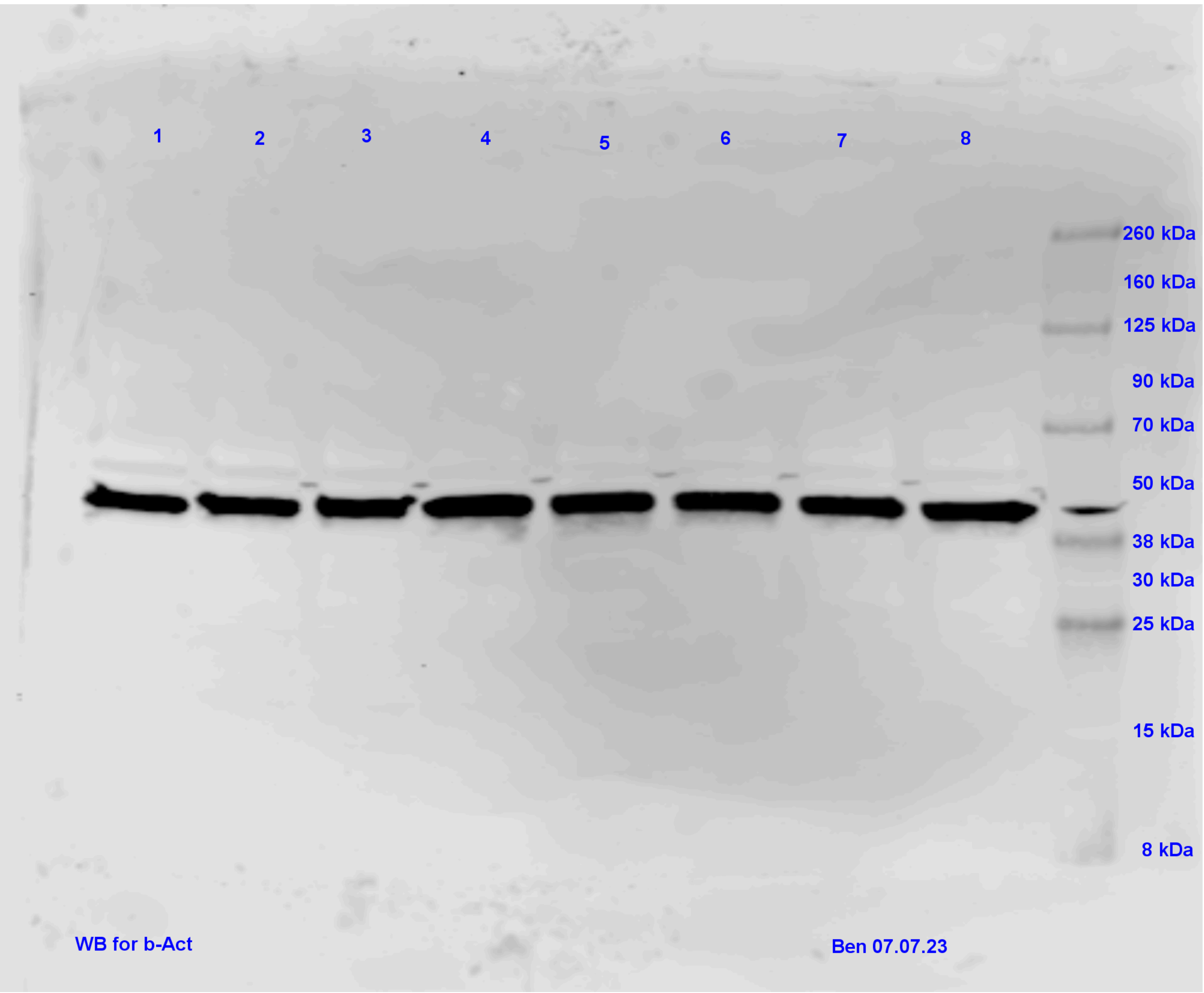

1
2
3
4
5
6
7
8
260 kDa
160 kDa
125 kDa
90 kDa
70 kDa
50 kDa
38 kDa
30 kDa
25 kDa
15 kDa
8 kDa
WB for b-Act
Ben 07.07.23

Figure 7E, part 1/2

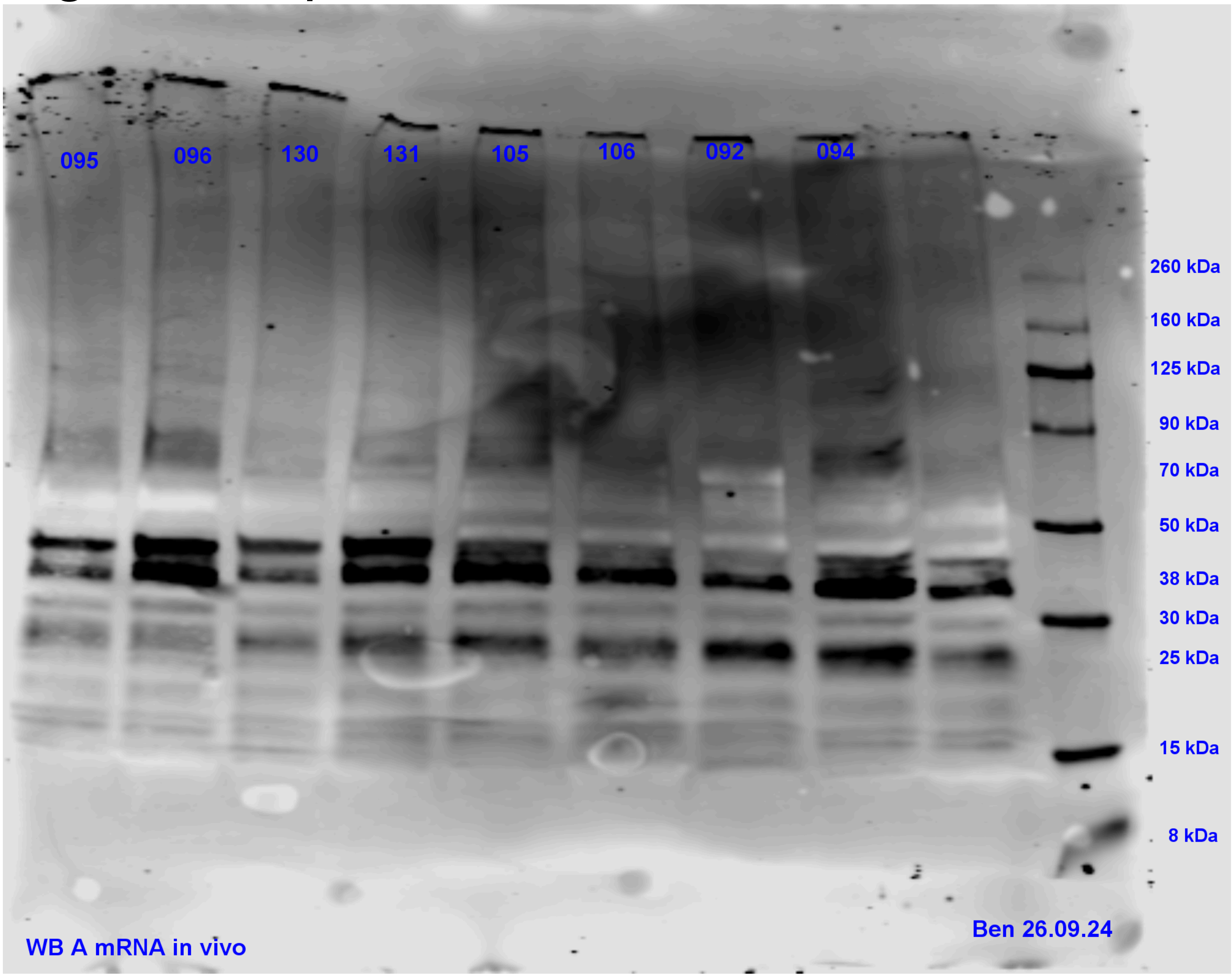

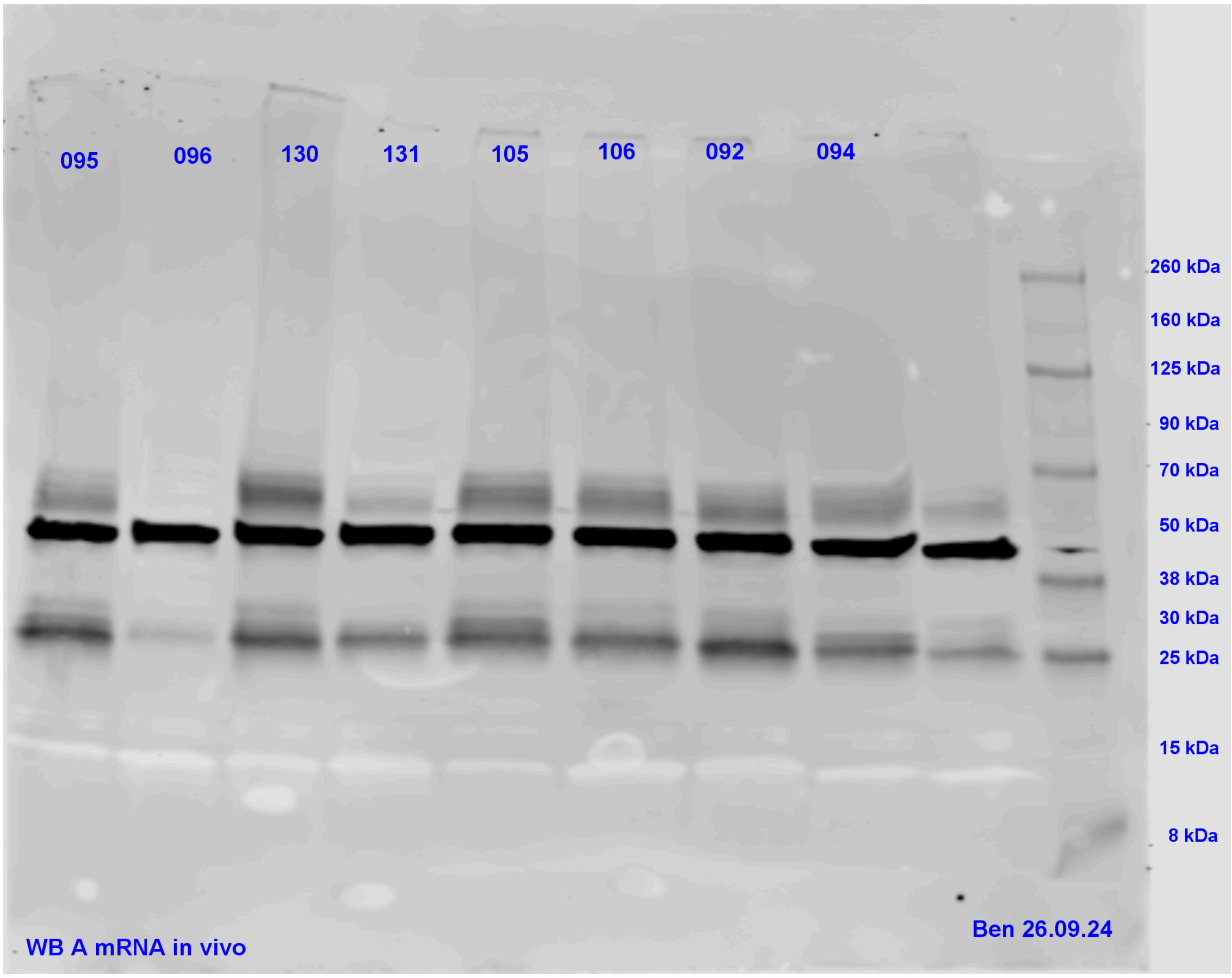

Figure 7E, part 2/2

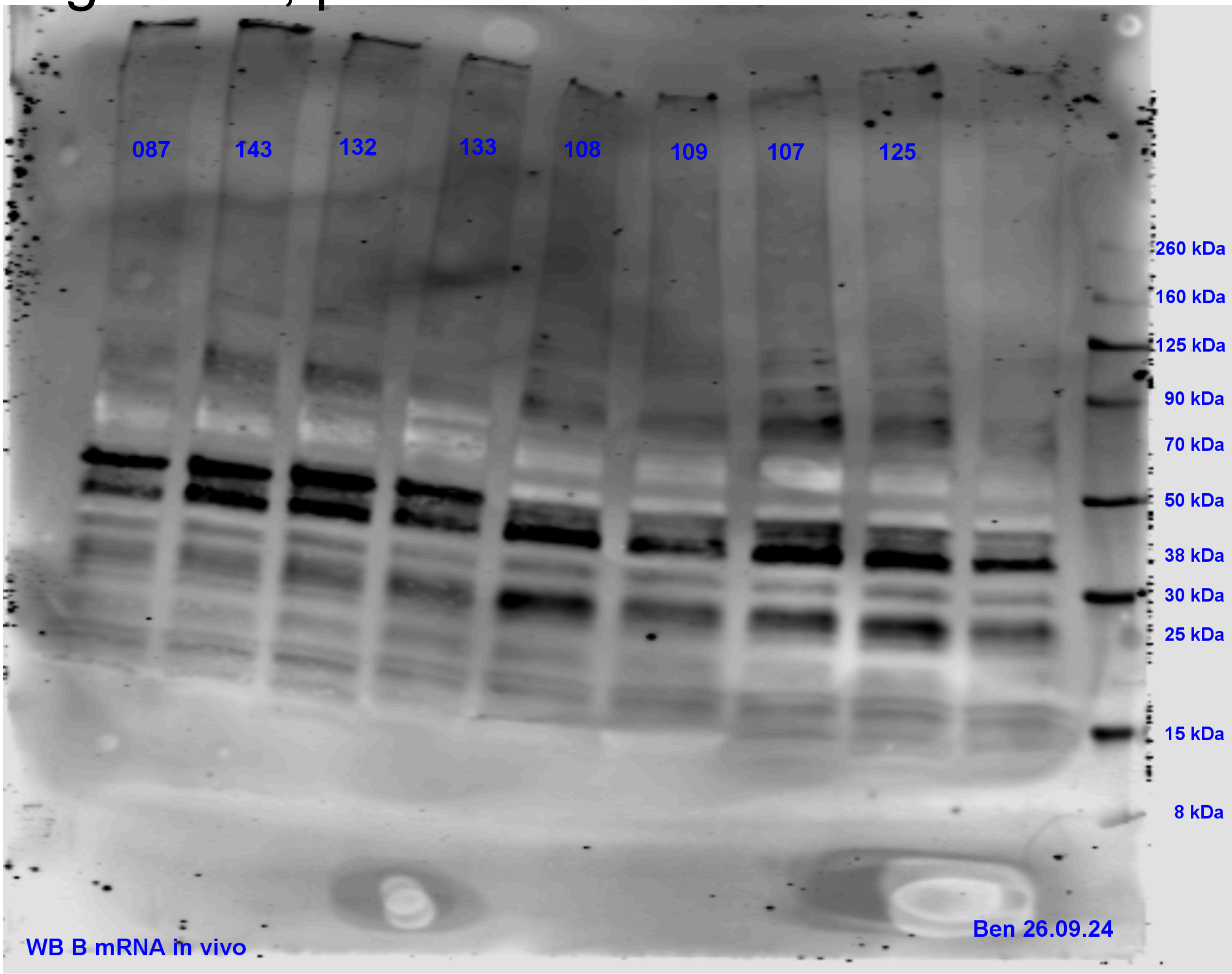

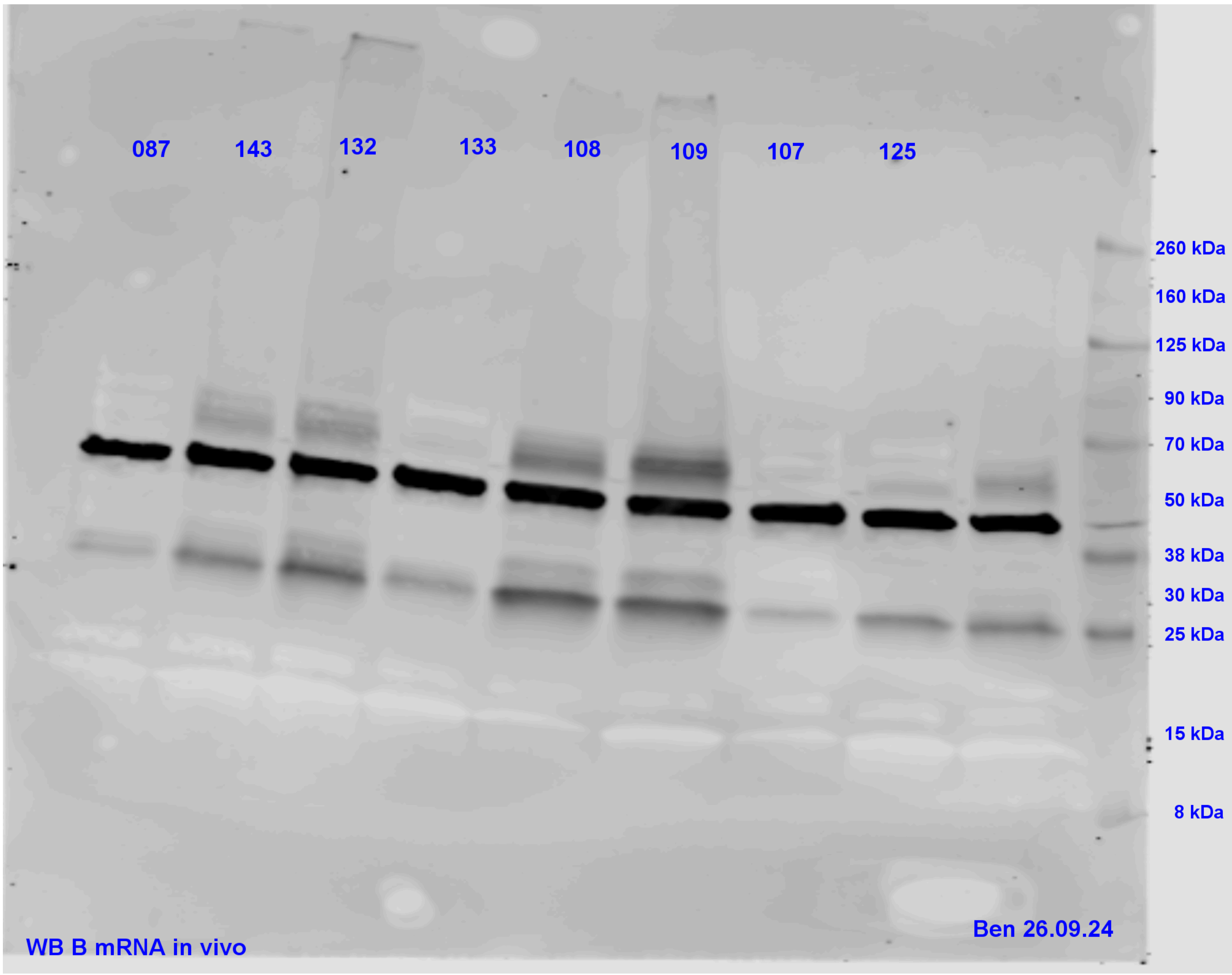

Supplementary Figure 1A

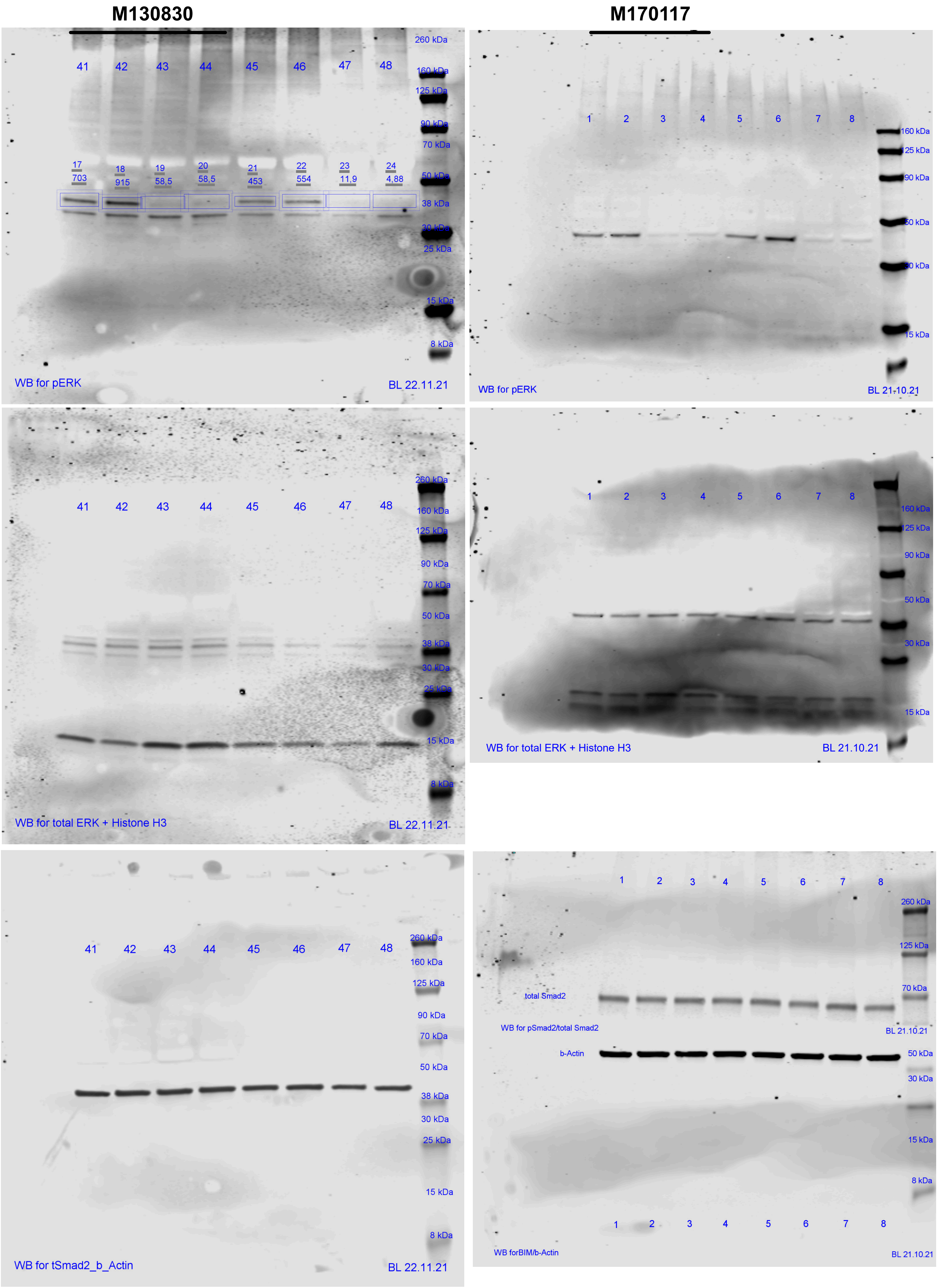

Supplementary Figure 1A

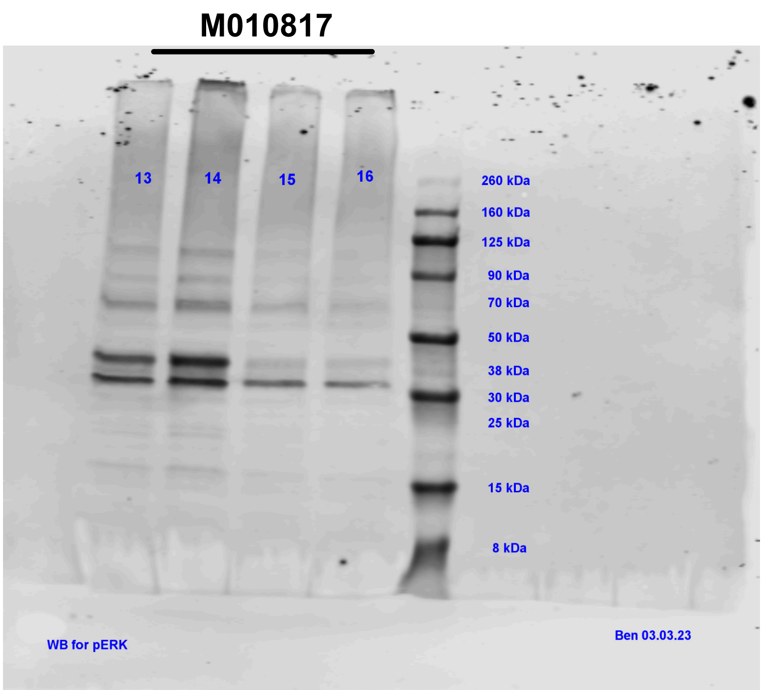

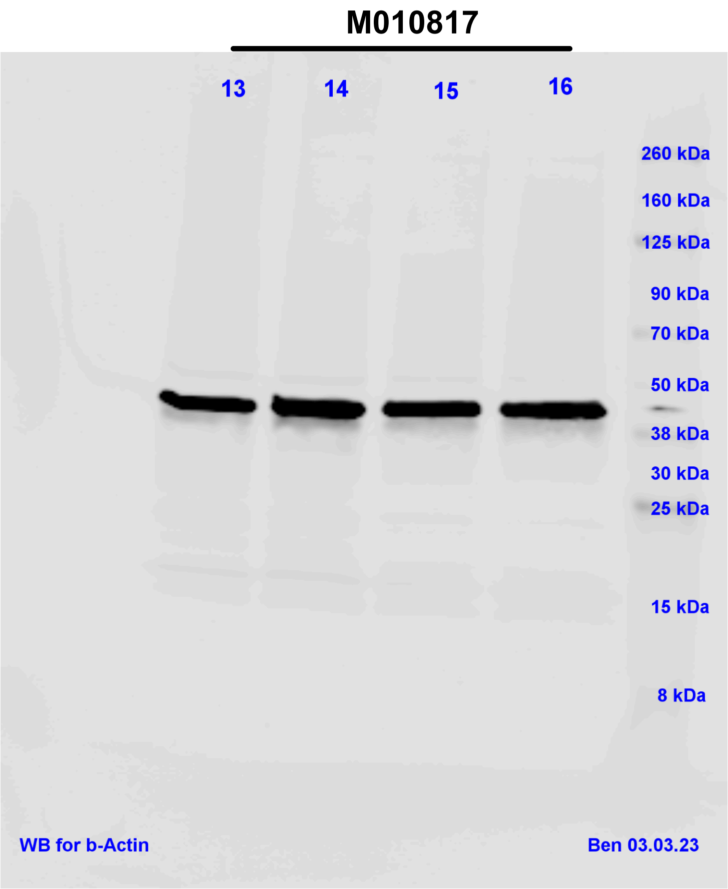

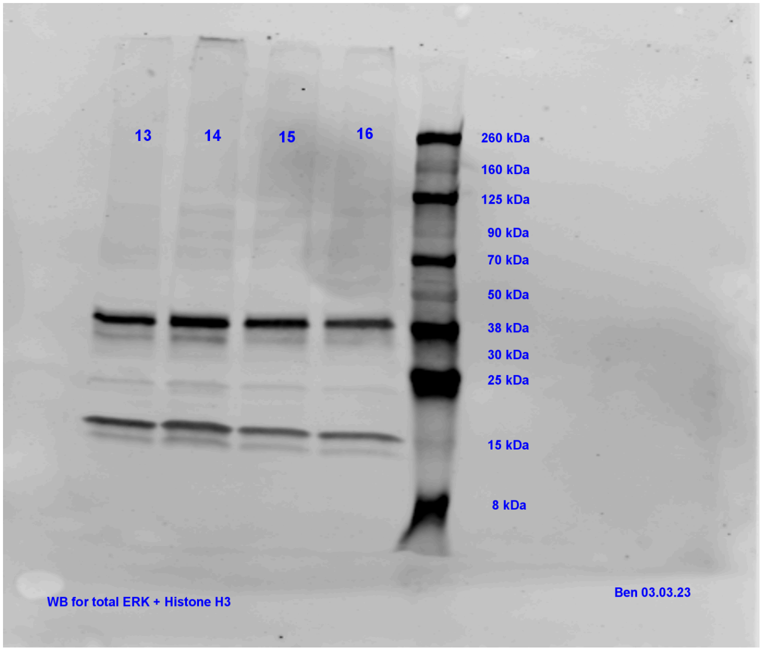

Supplementary Figure 1A + E - G

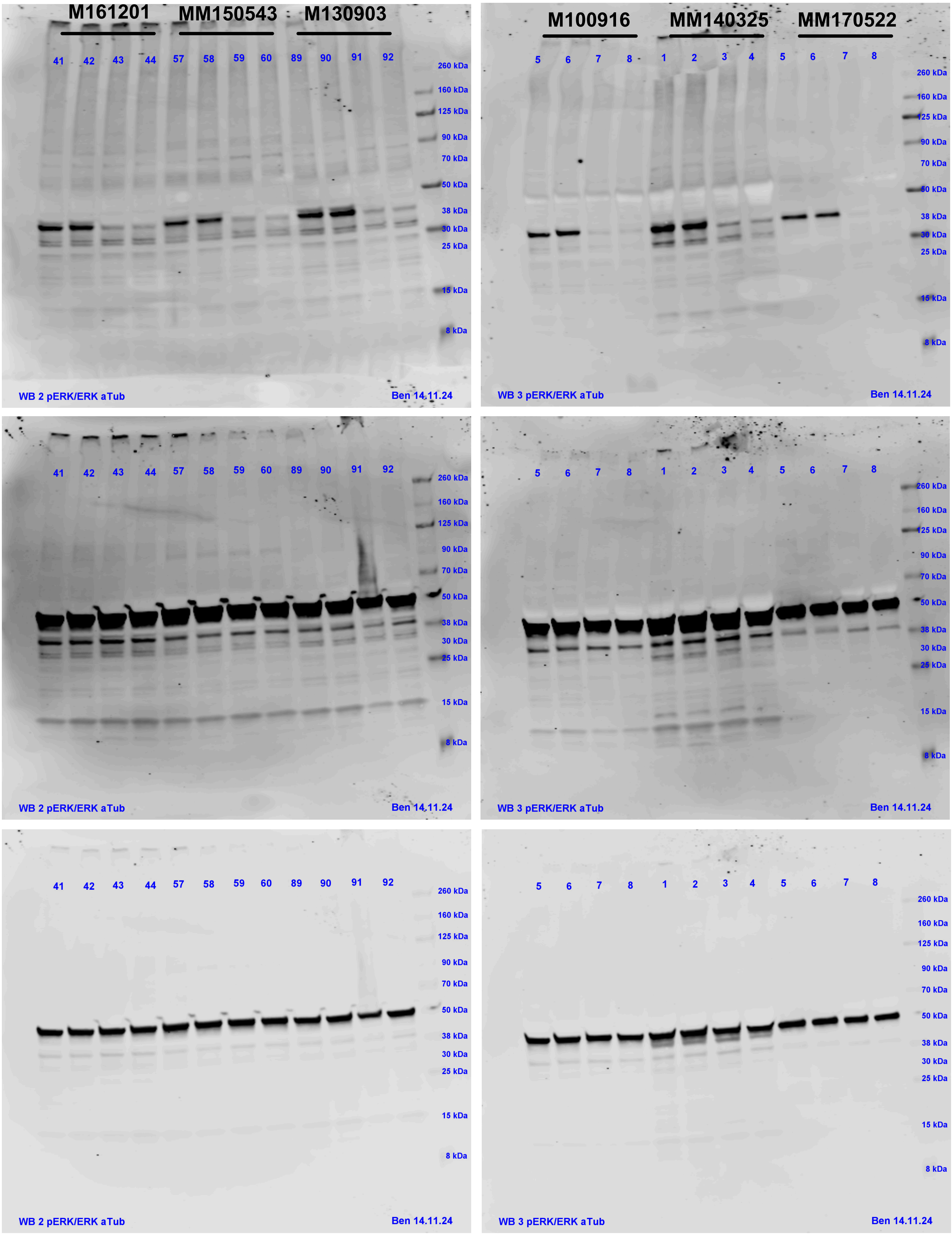

REP1

Supplementary Figure 2D

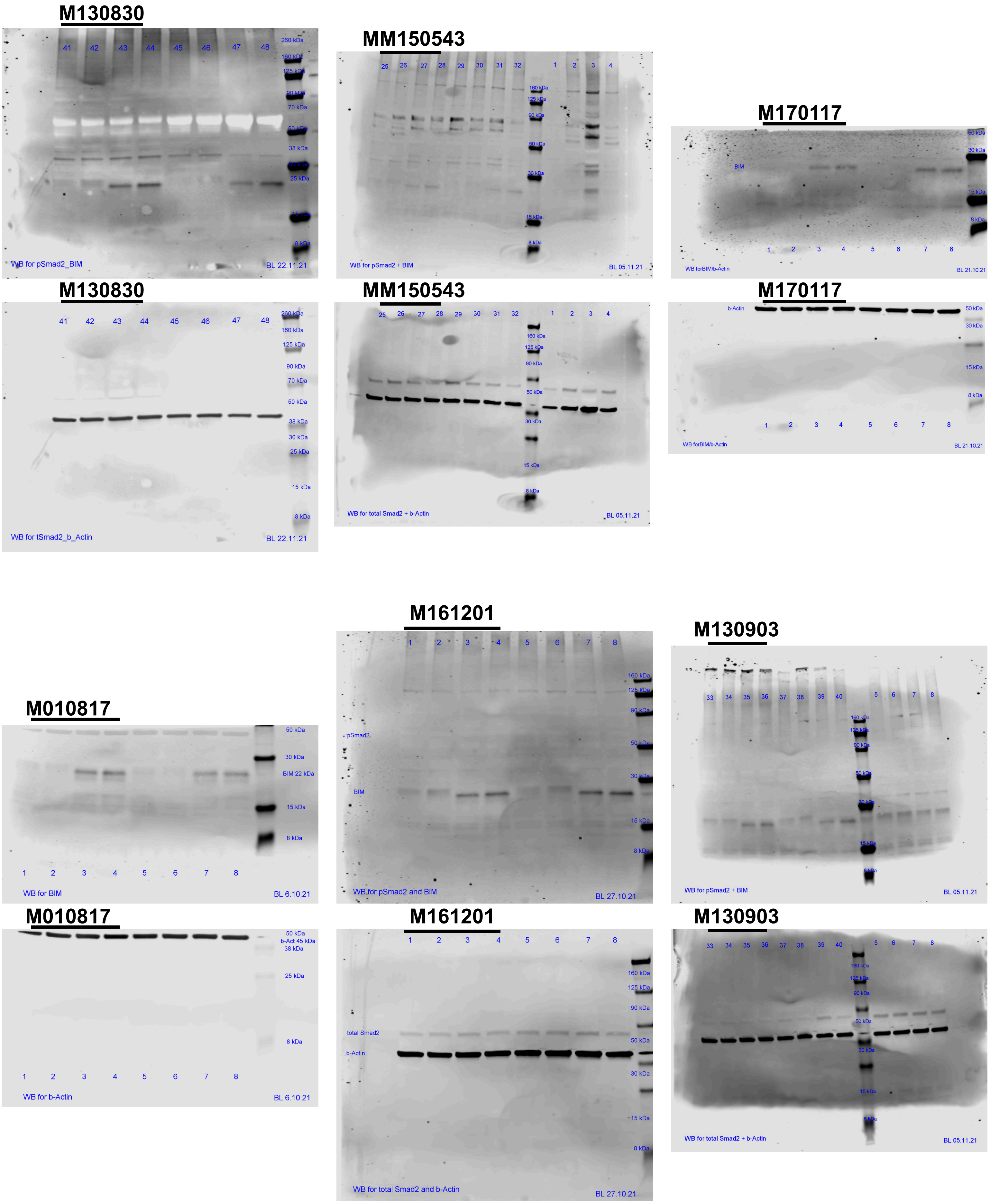

Supplementary Figure 2D

REP2

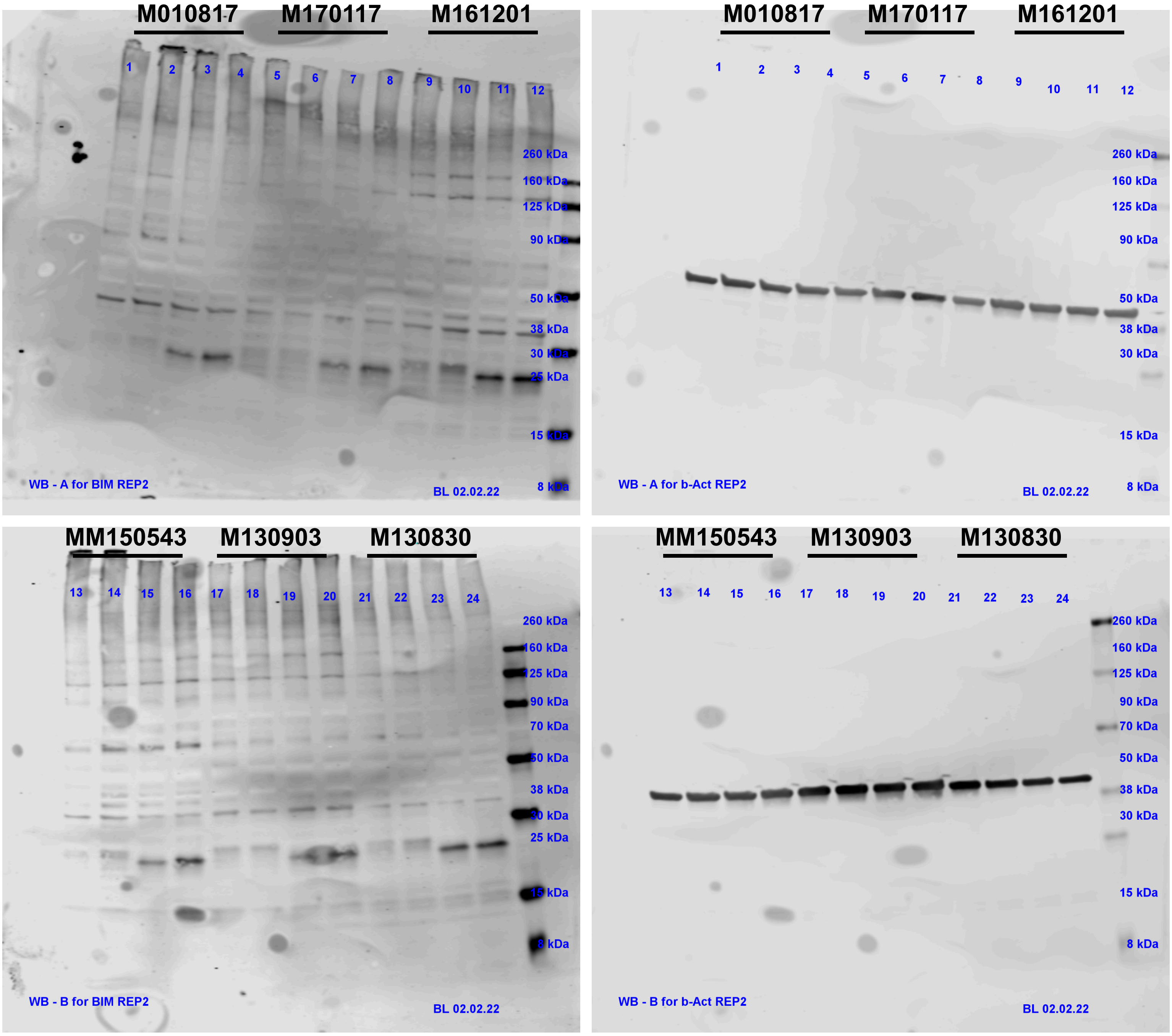

Supplementary Figure 2D

REP3

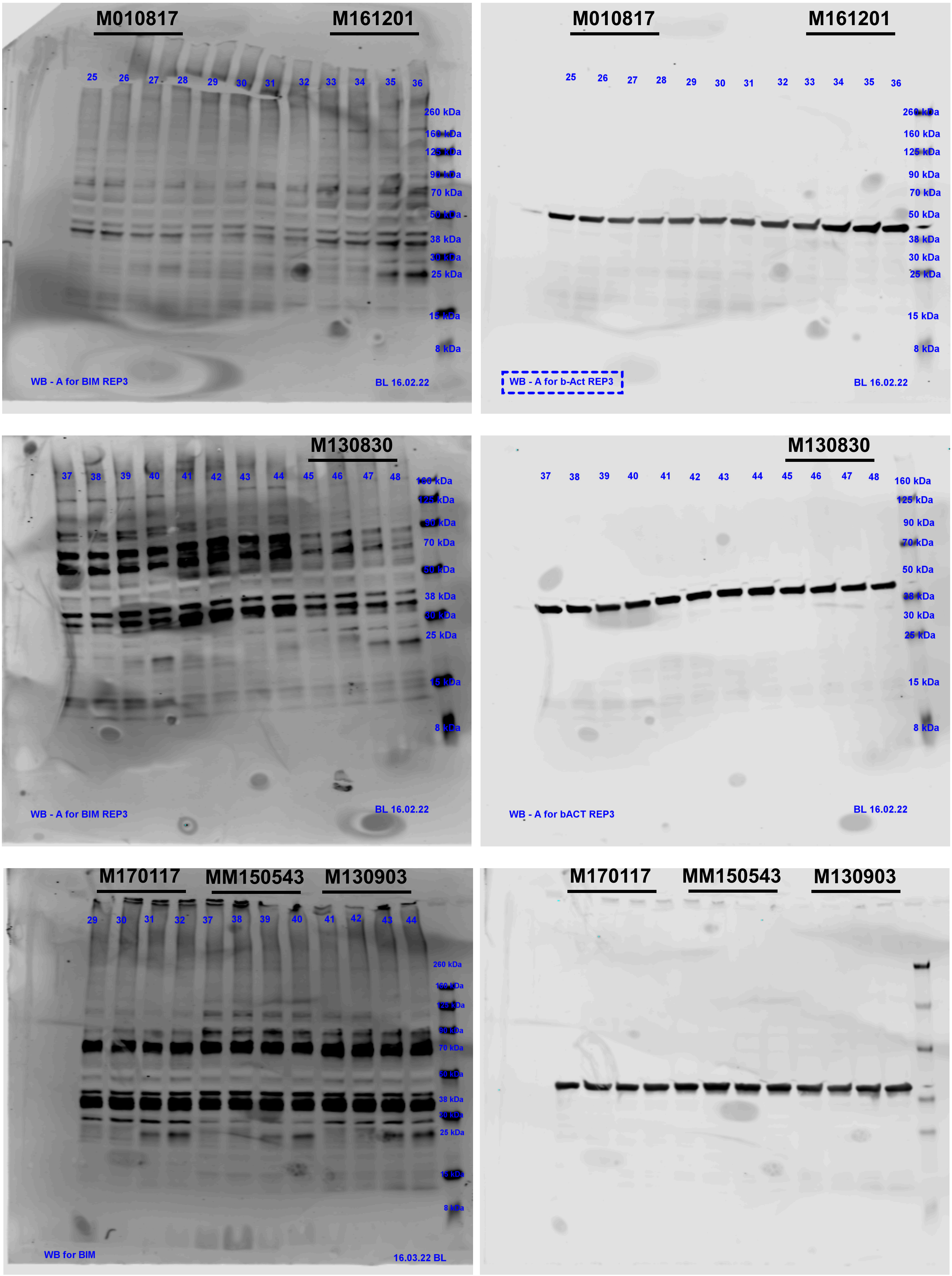

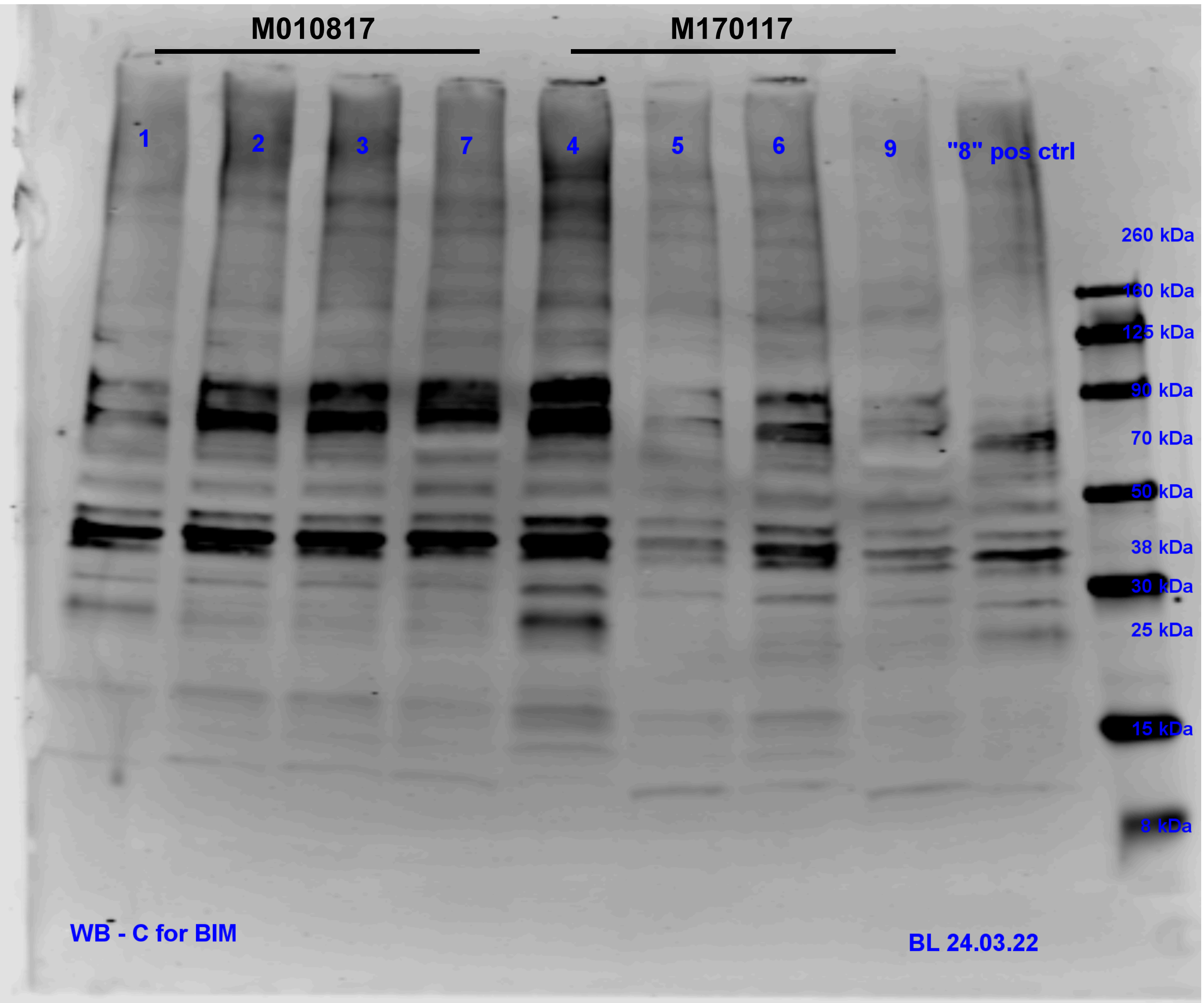

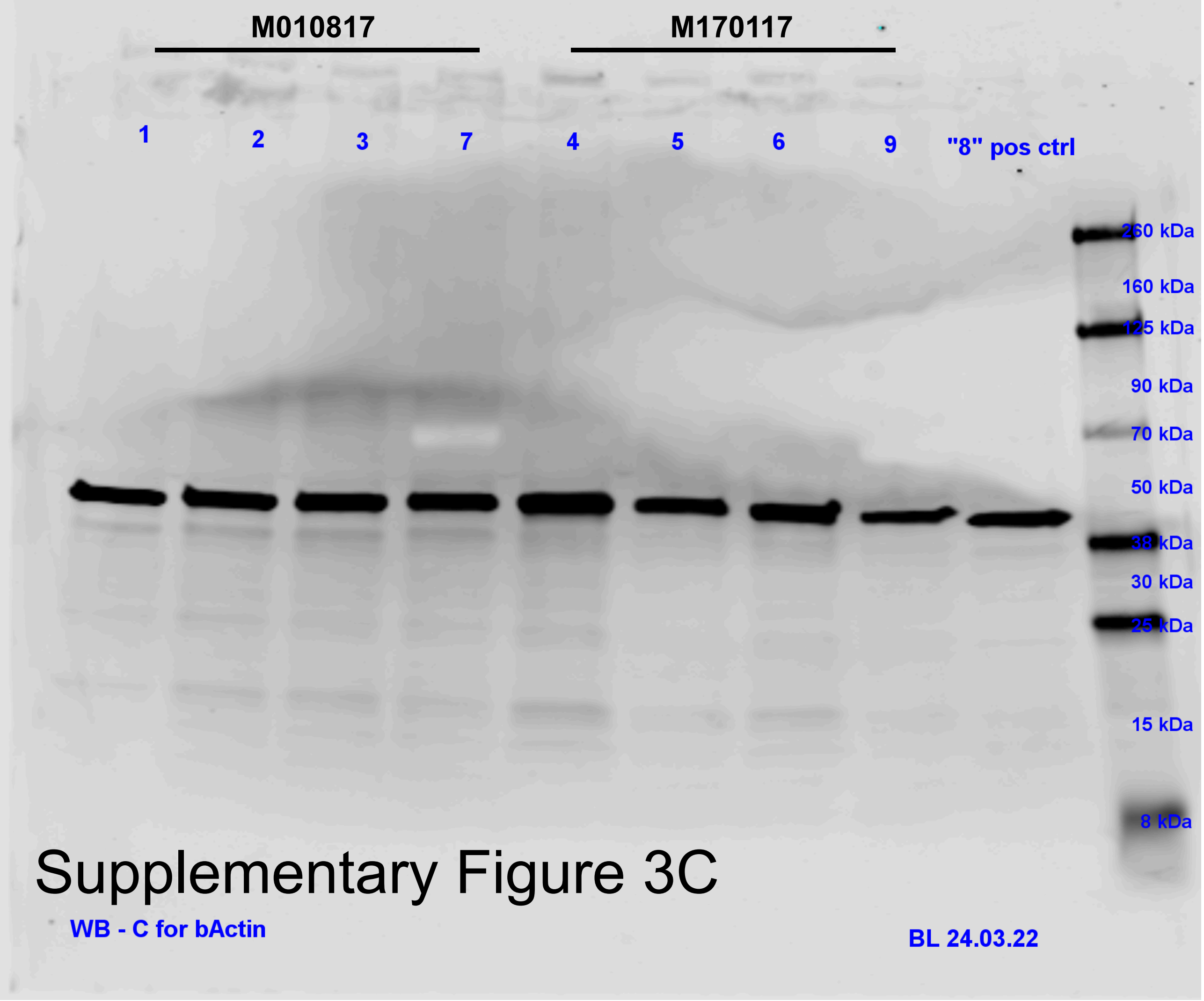

Supplementary Figure 3C

Supplementary Figure 3D

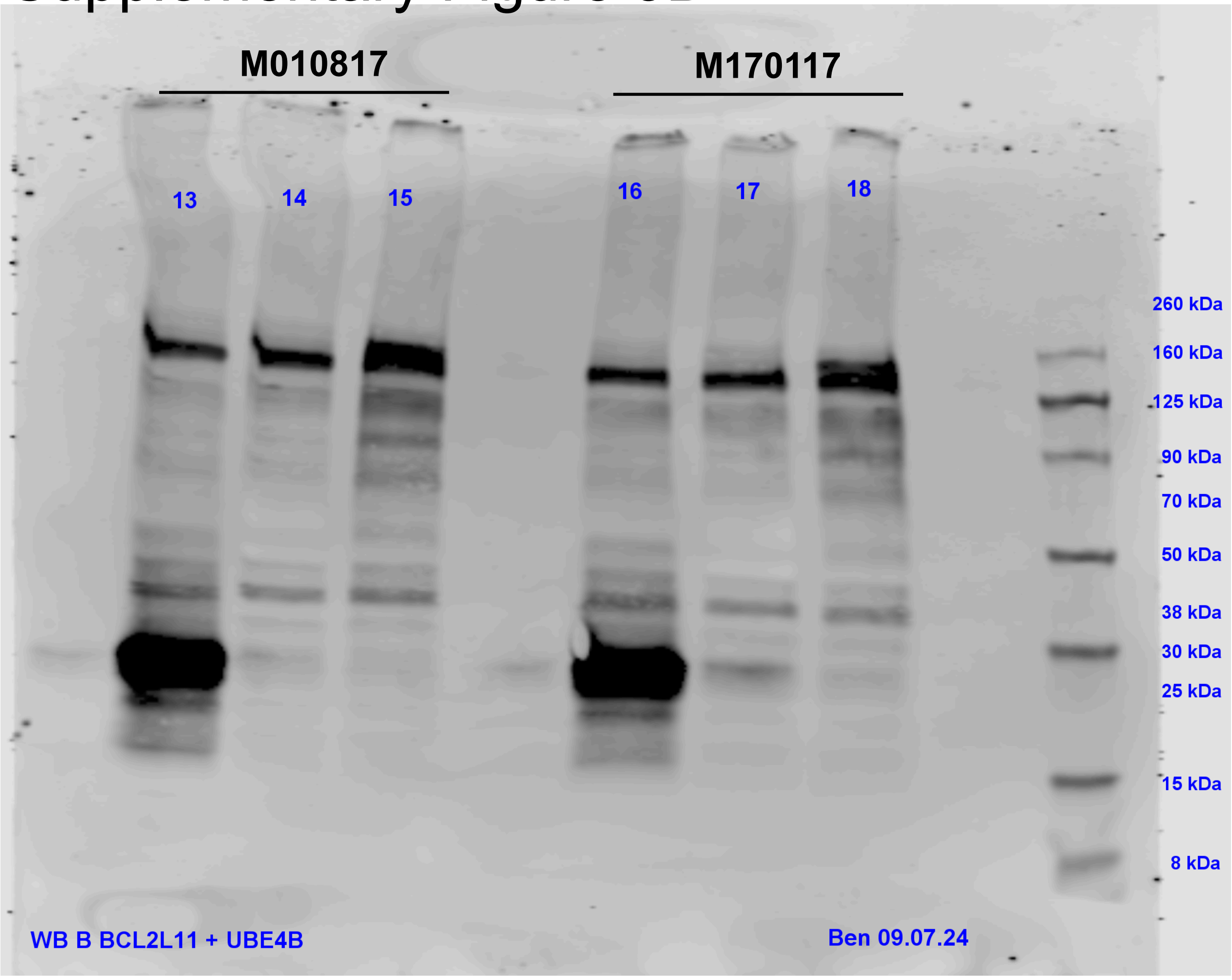

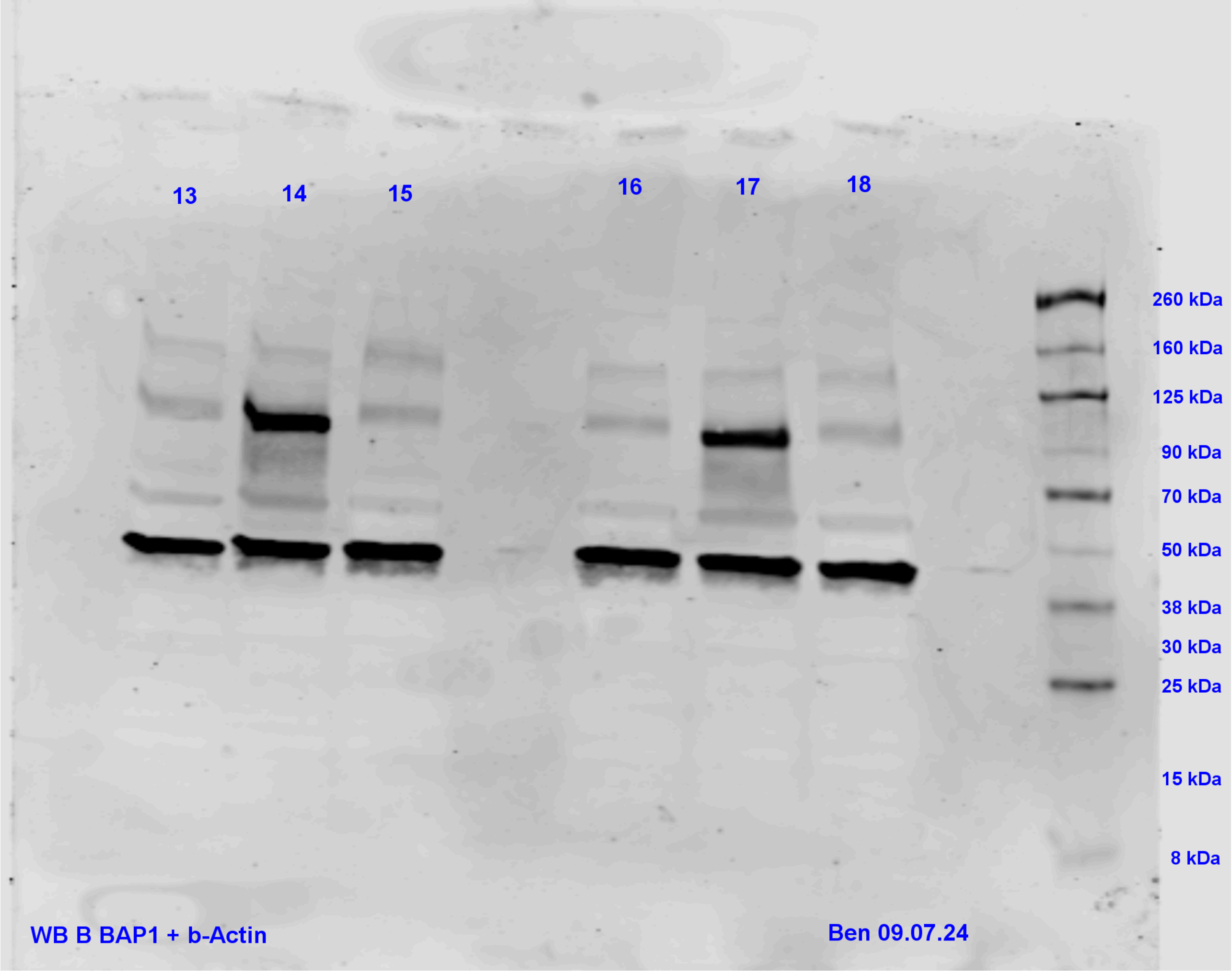

Supplementary Figure 4B

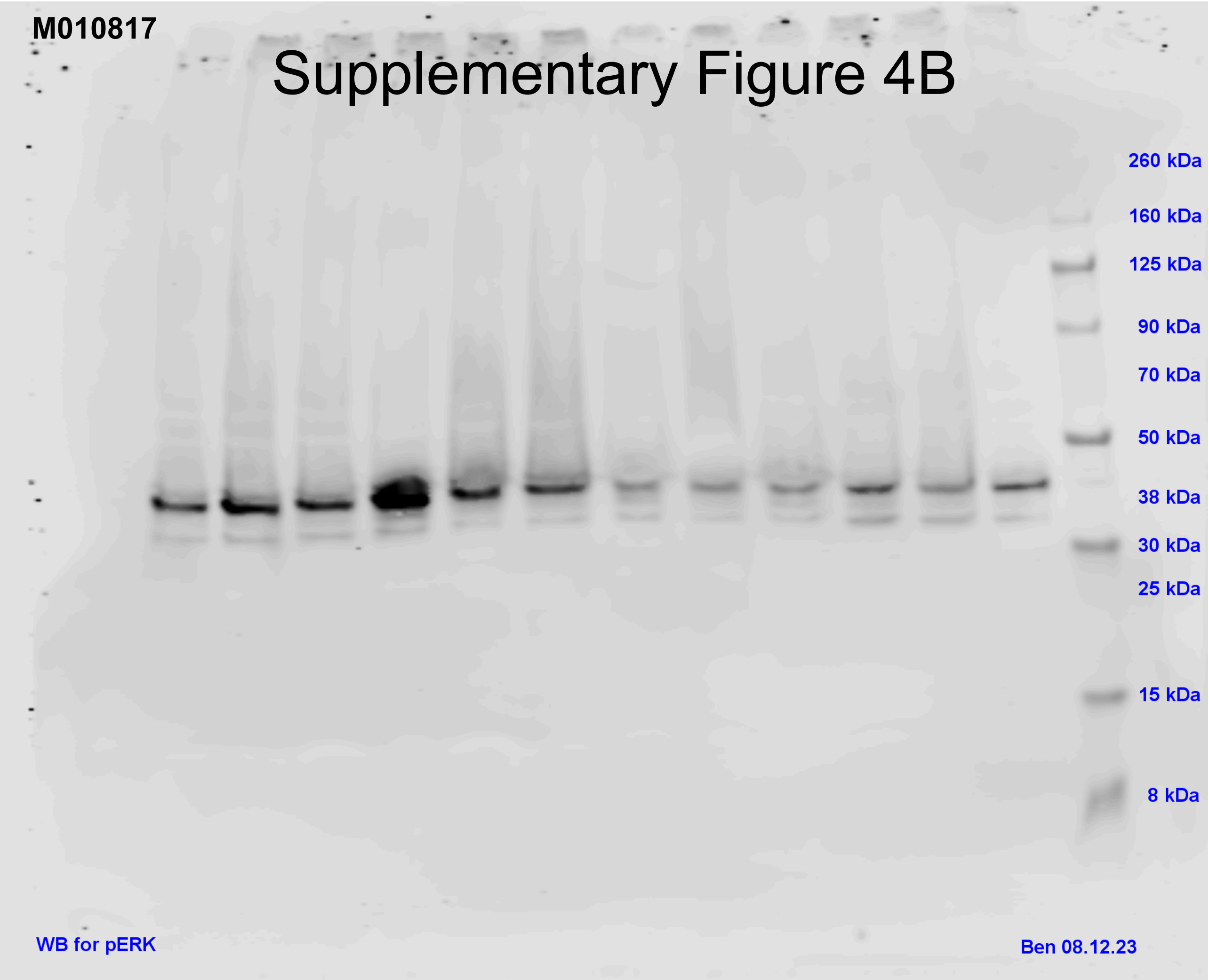

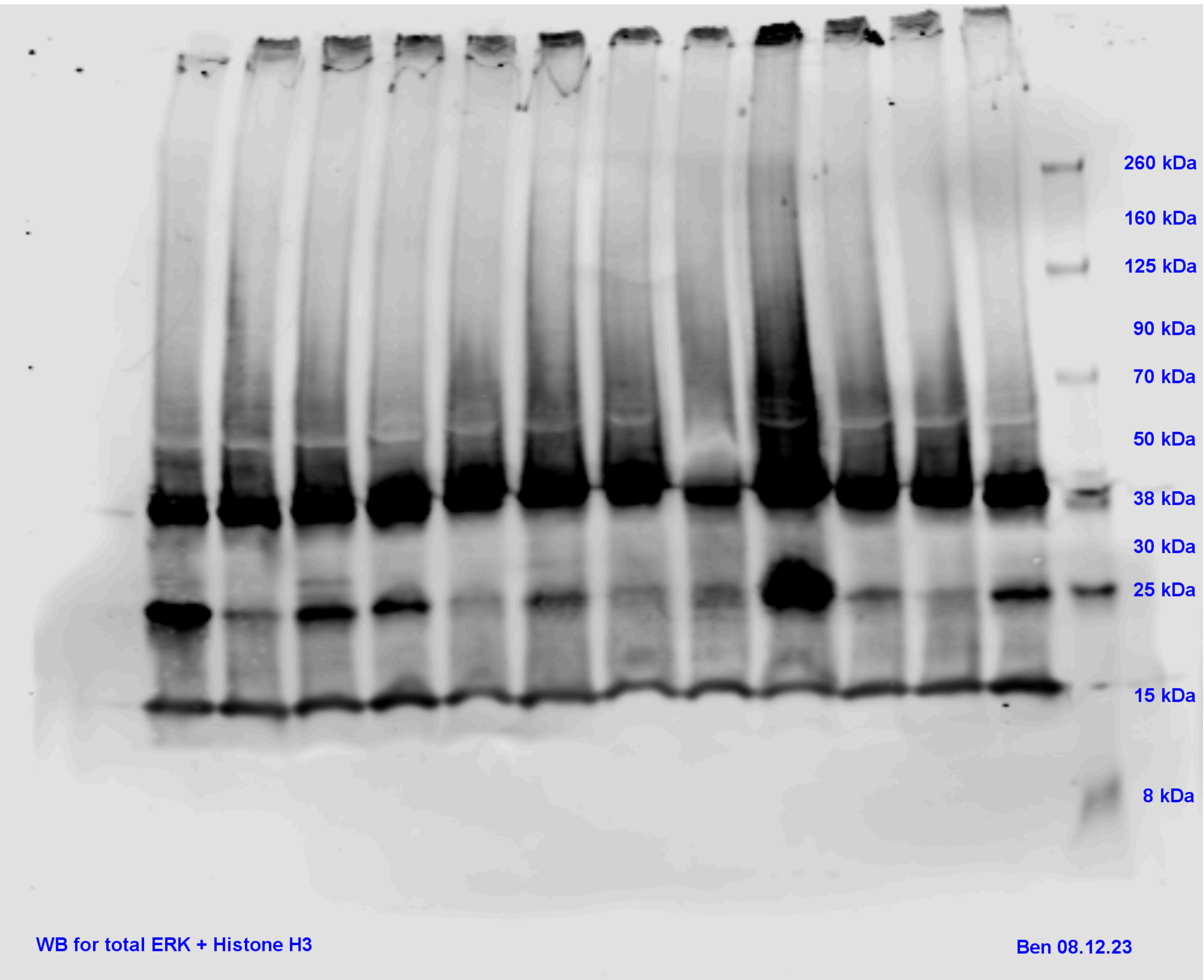

Supplement: Supplementary file 7 — Full size western blots [file 41419_2024_7305_MOESM7_ESM.pdf]
